# Rosuvastatin Prevents the Exacerbation of Atherosclerosis in Ligature-Induced Periodontal Disease Mouse Model

**DOI:** 10.1038/s41598-020-63350-8

**Published:** 2020-04-14

**Authors:** Jin Sook Suh, Sung Hee Lee, Zachary Fouladian, Jae Young Lee, Terresa Kim, Mo K. Kang, Aldons J. Lusis, Kristina I. Boström, Reuben H. Kim, No-Hee Park

**Affiliations:** 1 The Shapiro Family Laboratory of Viral Oncology and Aging Research, UCLA School of Dentistry, 10833 Le Conte Ave, Los Angeles, CA USA; 2 Department of Medicine, David Geffen School of Medicine at UCLA, 10833 Le Conte Ave, Los Angeles, CA USA; 3 UCLA Jonsson Comprehensive Cancer Center, 10833 Le Conte Ave, Los Angeles, CA USA

**Keywords:** Interventional cardiology, Chronic inflammation

## Abstract

Periodontitis is a local and systemic inflammatory condition and a risk factor of atherosclerosis, but no studies investigated the effect of a statin on atherogenesis affected by severe periodontitis. In this study, we investigated the effect of rosuvastatin (RSV) on atherogenesis in Apolipoprotein E-deficient mice receiving silk ligature placement around the maxillary second molars. Mice with the ligature placement developed severe periodontitis and vascular inflammation. RSV significantly inhibited the development of periodontitis and vascular inflammation and remarkably blocked the increased lipid deposition and the atherogenic gene expression in the arterial wall and aortic sinus induced by severe periodontitis. To understand the mechanistic effect of RSV on periodontitis-associated atherogenesis, we investigated the *in vitro* effect of RSV on various effect of TNF-α, a major proinflammatory cytokine for periodontitis and atherogenesis. We found that RSV notably inhibited the TNF-α-induced osteoclast formation, endothelial cell phenotypic changes, foam cell formation, and the expression of CD47 and other oncogenes in arterial smooth muscle cells. Taken together, our study indicates that RSV prevents the exacerbation of atherosclerosis induced periodontitis by inhibiting local, systemic and vascular inflammation, as well as the expression of CD47 from arterial smooth muscle cells in mice.

## Introduction

Periodontal disease comprises a wide range of local inflammatory conditions that affect the supporting structures of the teeth including gingiva, bone and periodontal ligament^1^. Periodontal disease also induces systemic inflammation, resulting in aggravation of various systemic conditions such as diabetes mellitus, neurodegenerative diseases and atherosclerosis^2^.

Atherosclerosis is a degenerative vascular disease accompanying chronic and progressive inflammatory conditions, which results in fatal consequences such as myocardial infarction and stroke due to the development of atherosclerotic plaque^3^. Although hypercholesterolemia is known as a major risk factor for the development of atherosclerosis, recent studies indicate that vascular inflammation coupled with dyslipidemia may be the etiology of atherogenesis as vascular inflammation induces vascular endothelial cell dysfunction, an early critical step of atherogenesis. Also, the inflammation causes atheroma progression and complications by inducing the proliferation and migration of vascular smooth muscle cells (VSMCs), resulting in thrombosis^4^.

Vascular endothelial cells, under normal conditions, function as a barrier that regulates molecular or cellular entry into the intima of blood vessels. However, when exposed to inflammatory cytokines or injury, the endothelial cells express membrane adhesion molecules which allow circulating monocytes to attach to endothelial cells and enter into the intima. Furthermore, the vascular inflammation induces proliferation of VSMCs, leading to intimal hyperplasia and extracellular matrix deposition^5^. Tumor necrosis factor-alpha (TNF-α), a secretory inflammatory cytokine expressed from macrophages and VSMCs, is one of the major proinflammatory cytokines associated with atherosclerosis and is known to promote proliferation and migration of VSMCs^6^.

Statins, hydroxymethyl glutaryl coenzyme A (HMG-CoA) reductase inhibitors, reduce low density lipoprotein-cholesterol (LDL-C) levels and effectively reduce the risk of atherosclerotic cardiovascular disease (CVD)^7^. In addition to their LDL-C lowering effects, statins also increase high density lipoprotein-cholesterol (HDL-C), decrease triglycerides (TG), and promote anti-inflammatory activities and pleiotropic positive effects in various systems, including the cardiovascular, immune, and skeletal systems^8,9^. In fact, statins reduce periodontal inflammation in humans^10^ and protect human endothelial cells from TNF-α-induced inflammation pathways, such as reactive oxygen species (ROS) formation, nuclear factor kappa-light-chain-enhancer of activated B cells (NF-κB), intercellular adhesion molecule (ICAM) and vascular cell adhesion molecule (VCAM) expression induced by TNF-α *via* extracellular signal-regulated kinase 5 (ERK5) activation^11^. Further, statins also protect endothelial progenitor cells from TNF-α-induced apoptosis^12^. The potency of the different statins vary, and newer agents (e.g., atorvastatin and rosuvastatin) appear to be more effective in lowering serum LDL-C levels than the previously introduced agents (e.g., simvastatin and pravastatin), in part, due to their ability to bind to hepatic HMG-CoA reductase with higher affinity and to inhibit the enzyme activity for a longer duration^13^. Among them, rosuvastatin (RSV) is the most potent hydrophilic statin with fewer side effects and longer terminal-lifetime than other common statins such as simvastatin and atorvastatin^14^. RSV also exerts a strong anti-inflammatory effect by inhibiting the c-Jun terminal kinase, the activation of nuclear factor kappa B (NF-κB) and the secretion of pro-inflammatory cytokines from macrophages^15^.

We and others have reported that severe periodontitis exacerbates atherogenesis *via* induction of systemic inflammation in Apolipoprotein E-deficient (*ApoE*^−/−^) mice^2,16^. In the present study, we investigated the effect of RSV on the features of atherosclerosis influenced by severe periodontitis in mice. To understand the mechanistic effect of RSV on periodontitis and periodontitis-associated atherosclerosis, we also investigated the *in vitro* effect of RSV on the phenotype of vascular endothelial cells, macrophages, and smooth muscle cells exposed to TNF-α, a proinflammatory cytokine associated with the development of periodontitis and atherosclerosis. Our study shows that RSV remarkably limits atherosclerosis induced by severe periodontitis in mice by suppressing the development of aberrant phenotype of endothelial cells, macrophages, and smooth muscle cells.

## Results

### RSV inhibited the severity of ligature-induced periodontitis in *ApoE*^−/−^ mice

**Figure 1:**
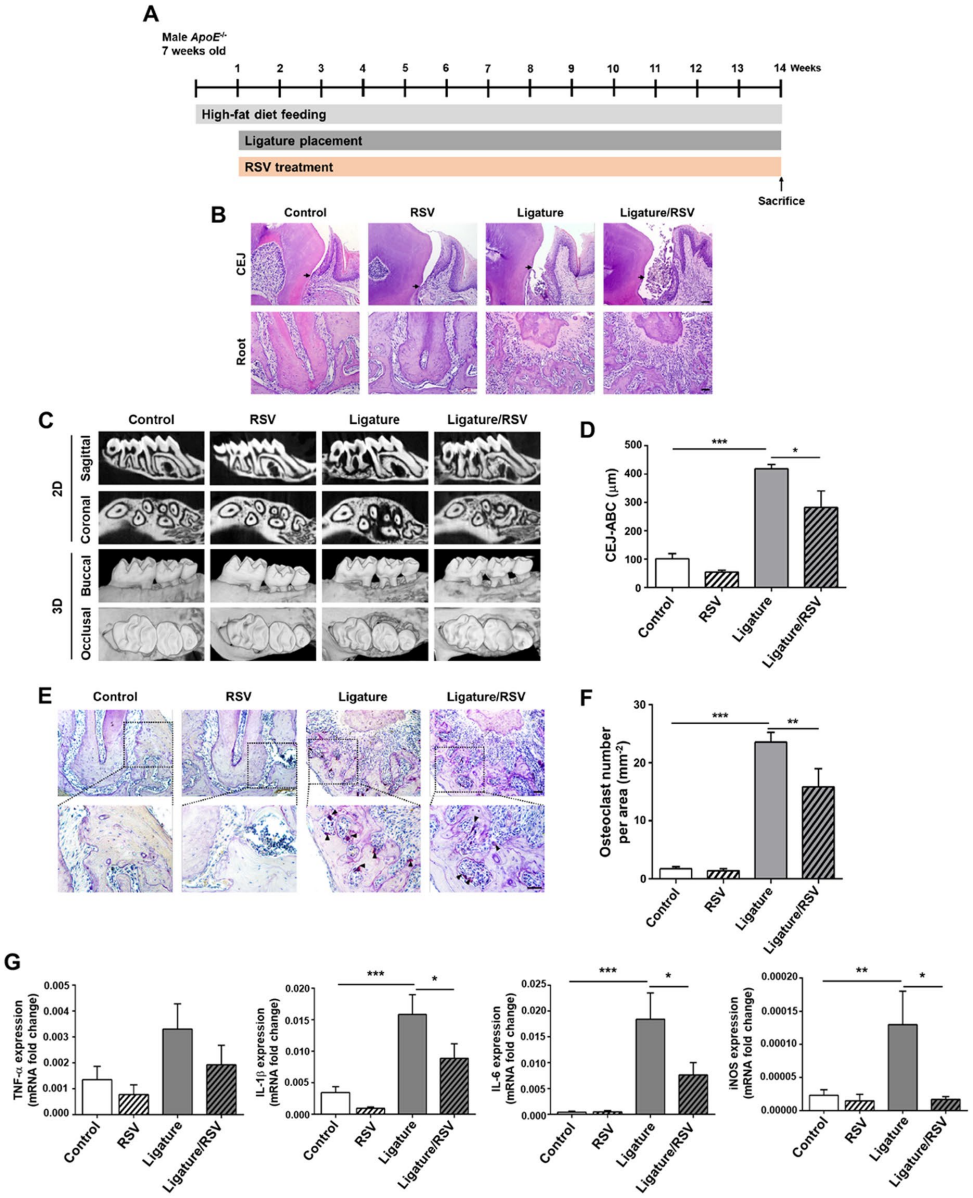
RSV reduced the severity of periodontal inflammation induced by ligature placement around the molars of *ApoE*^−/−^ mice. (**A**) Experimental design. Male *ApoE*^−/−^ mice fed with high fat diet (HFD) for one week were divided into four groups: (1) Control; (2) Mice fed with 0.005% RSV for 13 weeks; (3) Mice receiving ligature around the maxillary second molars for 13 weeks; and (4) Mice receiving with RSV and ligature. (**B**) Hematoxylin and Eosin (H&E) Staining of the periodontium of maxillary second molar. Scale bar: 50 μm. (**C**) Representative two dimensional or three dimensional μCT images of mouse maxilla. Scale bar: 1 mm. (**D**) Alveolar bone loss measured at the distobuccal (DB) root of the maxillary second molars from cemento-enamel junction (CEJ) to alveolar bone crest (ABC). (**E**) Tartrate-resistant acid phosphatase (TRAP)-stained tissues at the maxillary second molar areas. Arrows indicate osteoclasts. Scale bar: 50 μm. (**F**) Average number of TRAP^+^ osteoclasts per mm^2^ of alveolar bone. (**G**) Expression levels (determined by qRT-PCR) of pro-inflammatory cytokines (TNF-α, IL-1β, and IL-6) and iNOS at the palatal tissue of second molars. GAPDH served as loading control. *P < 0.05, **P < 0.01, and ***P < 0.001. Results represent the means ± SEM performed in triplicate.

To investigate the effect of RSV on the development of periodontitis, we utilized our previously established periodontitis model in *ApoE*^−/−^ mice^17^. Seven-week old male *ApoE*^−/−^ mice were fed with a high fat diet (HFD) for one week. Then, the mice were divided into four groups: (1) the Control group that received no RSV and no ligature; (2) the RSV group that received RSV administration but no ligature; (3) the Ligature group that received a ligature around maxillary second molars; and (4) the Ligature/RSV group that received both ligature placement and RSV administration. The ligature placement was performed by placing 6-0 silk suture ligatures around the 2nd maxillary molars (M2), and HFD and RSV administration were continued for an additional 13 weeks (Fig. 1A). The mice were sacrificed at the end of the experimental period to determine the severity of the periodontitis by histological examinations, alveolar bone loss, and gingival inflammation. The microscopic findings revealed that the ligature placement induced the loss of periodontal attachment as demonstrated by the degree of detachment of gingival tissue from the CEJ (Arrow, Fig. 1B), along with the severe alveolar bone loss measured by µCT scanning (Fig. 1C,D). RSV significantly reduced the alveolar bone loss induced by the ligature placement and also reduced the number of osteoclasts, an indication of alveolar bone resorption (Fig. 1E,F). To determine if RSV could inhibit osteoclast differentiation, we isolated bone marrow cells from wild-type male C57BL/6 J mice and induced osteoclast differentiation *ex vivo*. This was achieved by culturing the cells in the presence of mouse macrophage-colony stimulating factor (M-CSF) for 3 days, followed by exposing the cells to receptor activator of nuclear factor kappa-Β ligand (RANKL) and M-CSF for 0–3 days with or without RSV. Fully differentiated osteoclasts were detected as TRAP-positive multi-nucleated cells containing more than three nuclei. Although mono-nuclear TRAP-positive cells were formed in the presence of RSV, RSV treatment significantly inhibited multi-nuclear osteoclast formation induced by RANKL (see Supplementary Fig. S1). Consistently, expression of the RANKL-induced osteoclast markers, such as matrix metallopeptidase 9 (MMP9), Tartrate-resistant acid phosphatase type 5 (ACP5), Cathepsin K (CtsK) and Nuclear factor of activated T-cells, cytoplasmic 1 (NFATC1) were significantly decreased with RSV treatment (see Supplementary Fig. S1). These results suggest that the reduced alveolar bone loss by RSV administration is most likely due to the suppression of osteoclastogenesis by RSV administration. The ligature placement also notably increased the expression levels of critical pro-inflammatory cytokines, e.g., TNF-α, IL-1β and IL-6, and inducible nitric oxide synthase (iNOS) in palatal tissue around M2 (Fig. 1G). RSV also notably reduced the tissue levels of those pro-inflammatory cytokines which were enhanced by the ligature placement. These data indicate that RSV may reduce the severity of periodontal disease induced by the ligature placement in male *ApoE*^−/−^ mice via its anti-inflammatory activity. The effect of RSV on the severity of periodontitis in female *ApoE*^−/−^ mice induced by ligature placement was similar to that in male mice as shown in see Supplementary Fig. S2.

### RSV altered the serum lipid profile in *ApoE*^−/−^ mice and abolished the systemic inflammation induced by ligature placement

**Figure 2:**
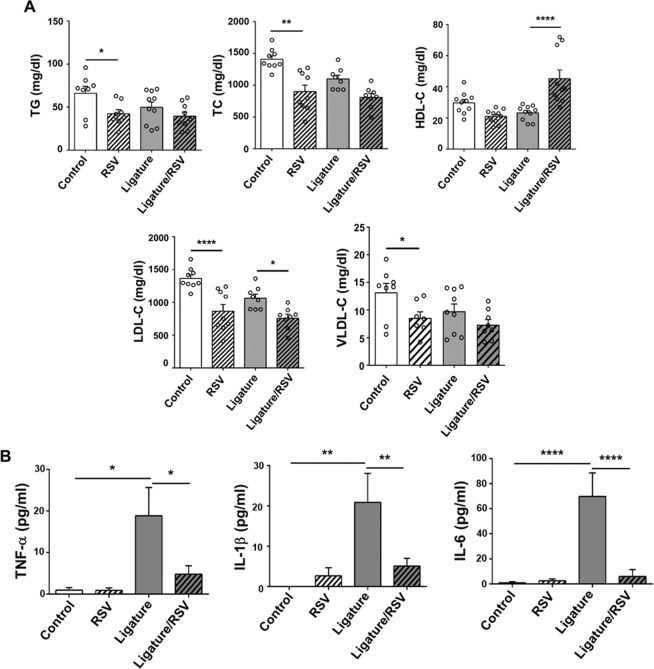
Ligature placement did not alter serum cholesterol profiles in *ApoE*^−/−^ mice, while RSV decreased notably alter the lipid profiles. Ligature placement notably increased the serum levels of pro-inflammatory cytokines, and RSV completely inverted such increases. Ligature placement also increased the TNF-α level from spleen, which was completely reversed by RSV. (**A**) Levels of Serum lipid; triglyceride (TG), total cholesterol (TC), high-density lipoprotein cholesterol (HDL-C), low-density lipoprotein cholesterol (LDL-C), and very low-density lipoprotein cholesterol (VLDL-C). (**B**) Levels of TNF-α, IL-1β and IL-6 from the mice sera by which were measured by using pre-coated ELISA plates. GAPDH served as loading control. *P < 0.05, **P < 0.01, and ****P < 0.0001. Results represent the means ± SEM performed in triplicate.

Long-term administration of RSV slightly, but significantly, reduced the serum levels of triglyceride (TG), total cholesterol (TC), LDL-C and VLDL-C in control *ApoE*^−/−^ male mice (Fig. 2A). However, RSV did not alter the lipid profile in female mice (see Supplementary Fig. S2). The ligature placement did not alter the serum lipid profile compared to that in mice without ligatures. Interestingly, RSV reduced the serum level of LDL-C, while it increased the level of HDL-C in male mice with the ligature placement (Fig. 2A). The serum levels of pro-inflammatory cytokines, such as TNF-α, IL-1β, and IL-6, were markedly increased by the ligature placement in both male and female mice, and such increases were almost completely prevented by the RSV administration (Fig. 2B; see Supplementary Fig. S2). The RSV administration also suppressed the enhanced level of TNF-α by ligature placement in spleen (see Supplementary Fig. S3). Furthermore, RSV administration not only altered the serum lipid profile in both control mice and the mice with ligature placement, but it also completely prevented the systemic inflammation induced by the ligature placement. The effect of RSV on the systemic inflammation caused by the ligature placement in female mice was similar to that in male mice, although the lipid profile in the control and mice with the ligature placement was not altered in female mice (see Supplementary Fig. S2). These data indicate that the anti-inflammatory effect of RSV might not be related to its lipid lowering effect in *ApoE*^−/−^ mice.

### Ligature-induced periodontitis markedly enhanced arterial lipid deposition in *ApoE*^−/−^ mice, and RSV negated this exacerbation

**Figure 3:**
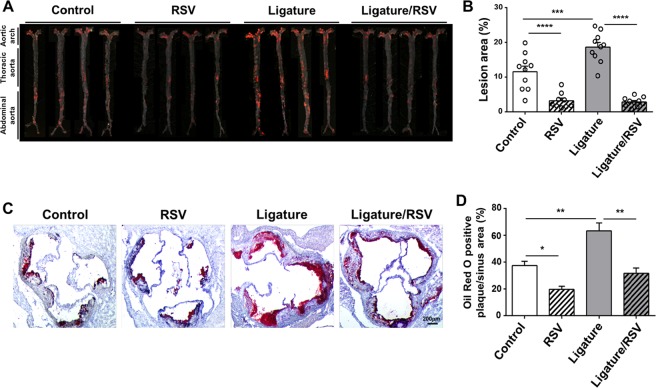
Ligature placement significantly increased the lipid deposition on the arterial wall and aortic root, and RSV almost completely blocked Ligature-induced lipid deposition. (**A**) Photographs of mice aortas from the *en face* preparation after staining with Sudan IV (10 mice per group). (**B**) Quantification of areas stained by Sudan IV. (**C**) Representative examples of cross sections from Oil Red O-stained aortic root (10 mice per group). (**D**) Quantification of aortic root lesion area stained by Oil Red O. *P < 0.05, **P < 0.01, and ***P < 0.001 and ****P < 0.0001. Results represent the means ± SEM performed in triplicate.

Lipid accumulation and the deposition of collagen and calcium in the arterial wall are characteristics of advanced atherosclerosis^18^. Therefore, we assessed the status of lipid, collagen, and calcium salt deposition in the arterial wall to examine whether the ligature placement caused advanced atherosclerosis. *En face* analysis demonstrated that minor to moderate lipid and collagen deposition in the arterial wall of control male mice fed with a HFD for 14 weeks, which was significantly suppressed by RSV administration (Fig. 3) similar to a previous report^19^. However, the amount of lipid deposition was not diminished by RSV in the control female mice (see Supplementary Fig. S2). Although we do not understand the reasons for this gender difference, it might be, in part, due to the differences in the serum lipid profiles in male and female mice receiving RSV administration.

**Figure 4:**
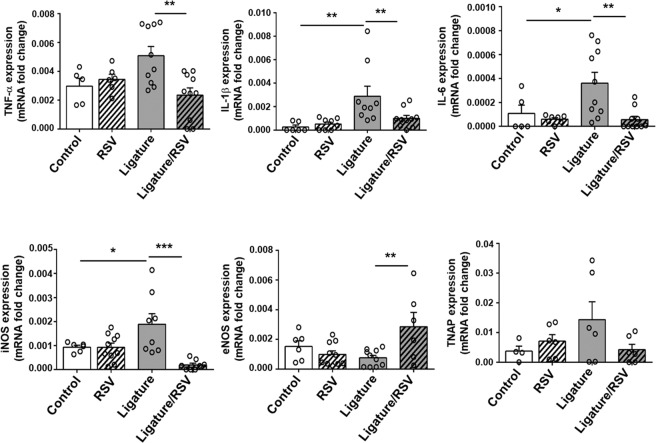
RSV inhibited ligature-induced expression of pro-inflammatory cytokines on aorta in mice. Levels of gene expression (TNF-α, IL-1β, IL-6, iNOS, eNOS and TNAP) from aortas determined by qRT-PCR. GAPDH served as loading control. **P < 0.05, **P < 0.01, and ***P < 0.001. Results represent the means ± SEM performed in triplicate.

Strikingly, the ligature placement noticeably induced high lipid accumulation in the arterial wall compared to the control mice, and this increase was significantly prohibited by RSV administration in both male and female mice (Fig. 3A,B; see Supplementary Fig. S2). Similarly, RSV administration also suppressed the dense lipid deposition found in the aortic roots (Fig. 3C,D) and brachiocephalic arteries (see Supplementary Fig. S4) of male mice. In addition, RSV administration significantly negated collagen deposition in the plague as demonstrated by Masson’s blue trichrome staining at the aortic roots (see Supplementary Fig. S5). Calcification of the plaques of the aortic root were also markedly suppressed by RSV administration (see Supplementary Fig. S5). Furthermore, the ligature placement notably increased an accumulation of cells stained with monocyte/macrophage antibody-2 (MOMA-2) antibodies in the aortic roots and brachiocephalic arteries, and RSV notably blocked these accumulations (see Supplementary Fig. S4). To determine the aortic inflammatory status, we performed qRT-PCR for determining the expression level of pro-inflammatory cytokines. As expected, the expression levels of TNF-α, IL-1β, IL-6, iNOS and tissue-nonspecific alkaline phosphatase (TNAP) were significantly increased with the ligature placement, but the increase was significantly negated by RSV (Fig. 4). These results suggest that RSV prevented the arterial inflammation and atherosclerotic development caused by the ligature-induced periodontitis. The effect of RSV on lipid deposition and vascular inflammation caused by the ligature placement in female mice was similar to that in male mice (see Supplementary Fig. S2). These data, along with the effect of lipid profile, indicate that RSV significantly inhibits atherosclerosis exacerbated by periodontitis beyond its lipid lowering effect in *ApoE*^−/−^ mice.

### RSV inhibited the adhesion of monocytes to endothelial cells induced by TNF-α and suppressed the formation of foam cells from macrophages *in vitro*

**Figure 5:**
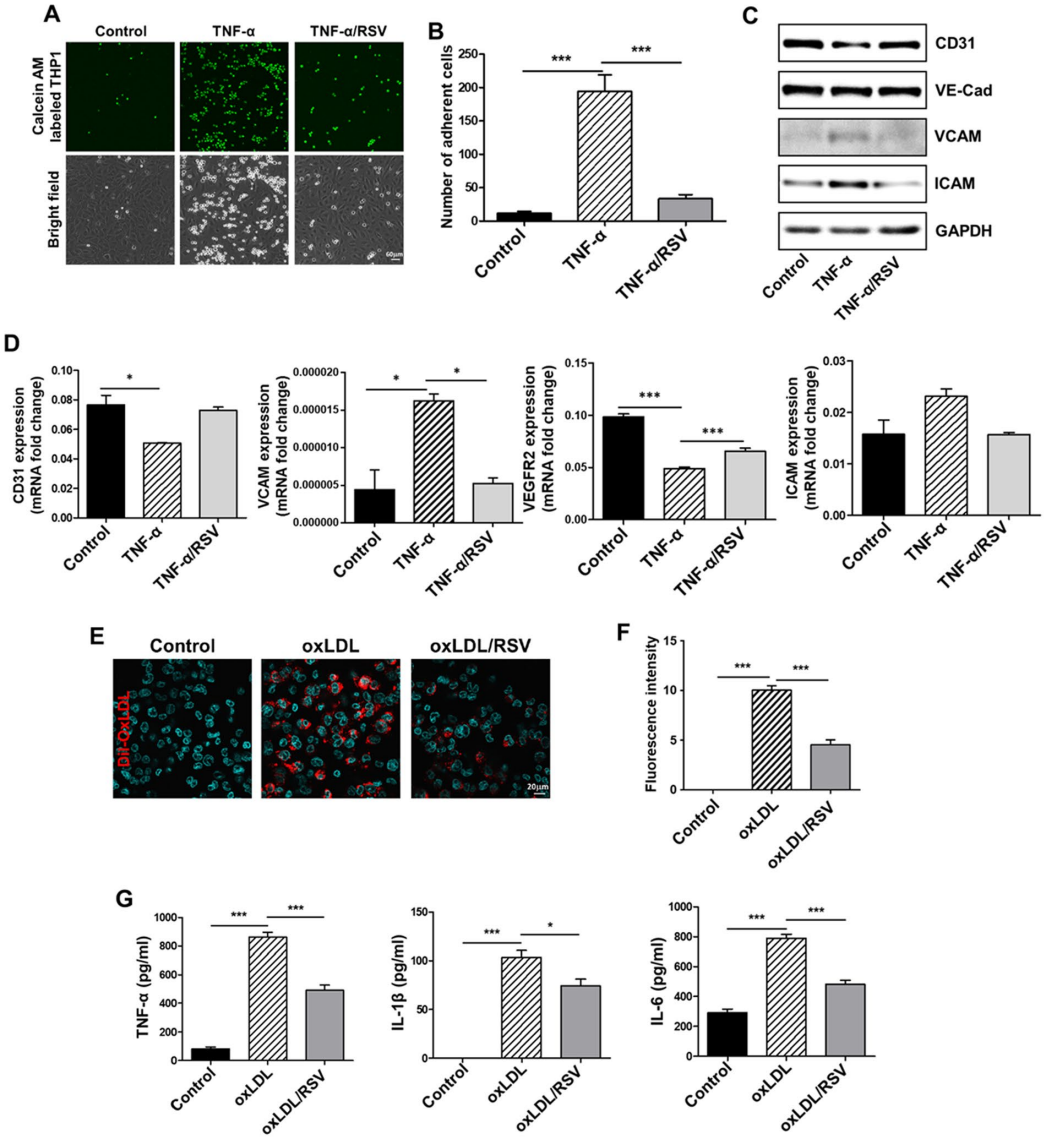
RSV suppressed the TNF-α-induced adhesion of human vascular endothelial cells (HUVECs) to human monocytes by hampering the expression of adhesion molecules from HUVECs. RSV also inhibits TNF-α-induced foam cell formation from macrophages. (**A**) Adhesion of fluorescence-tagged THP1 human monocytes to HUVECs exposed to 50 ng/ml TNF-α with/without 10 μM RSV. THP1 cells were labeled with Calcein AM and placed on three groups of HUVECs monolayer for 0.5 h. (**B**) Quantification of attached Calcein AM-labeled THP1 cells. (**C**) Levels of protein expression (CD31, VE-Cad, VCAM and ICAM) from aortas determined by Western blot. GAPDH served as loading control. (**D**) Levels of mRNA expression (CD31, VCAM, VEGFR2 and ICAM) from HUVECs determined by qRT-PCR. GAPDH served as loading control. (**E**) Determination of foam cell formation by using Dil-oxLDL (Red). Nuclei were counterstained with DAPI (Blue). (**F**) Quantification area stained by penetrating 50 μg/ml Dil-oxLDL (Red) to see foam cell formation. (**G**) Measurement of secretory proteins from THP1-derived macrophages. *P < 0.05, **P < 0.01, and ***P < 0.001. Results represent the means ± SEM performed in triplicate.

Many studies have indicated the critical role of TNF-α in the development of atherosclerosis^20–22^. As the severe periodontitis induced by the ligature placement resulted in very high serum level of TNF-α in mice and as many previous studies have indicated the important role of TNF-α on the development of atherosclerosis in mice^23,24^, we studied the mechanisms of periodontitis-induced atherosclerosis by investigating the role of TNF-α on various events of atherogenesis *in vitro* setting. Inasmuch as the initial step of atherogenesis is the binding of monocytes to arterial endothelial cells^25^, we first determined the effect of TNF-α on the binding of human monocytes (THP1) to human umbilical vessel endothelial cells (HUVECs) and the expression of adhesion molecules from HUVECs. When THP1 cells were co-cultured with the HUVECs in the presence of TNF-α, the number of THP1 attached to HUVECs notably increased (Fig. 5A,B). Moreover, the expression levels of the adhesion molecules from HUVECs, such as ICAM and VCAM, were enhanced by TNF-α as demonstrated by Western blot and qRT-PCR analyses (Fig. 5C,D). As shown in Fig. 5A–D, RSV prevented such *in vitro* effects of TNF-α. Furthermore, the Dil tagged-oxidized LDL (Dil-oxLDL) treatment on human macrophages demonstrated that RSV notably inhibited the formation of foam cells from macrophages (Fig. 5E,F). Moreover, the level of inflammatory cytokines (e.g., TNF-α, IL-1β, and IL-6) secreted from oxLDL-treated macrophages were highly enhanced, but this increase was significantly lessened in the presence of RSV (Fig. 5G). These data suggest that RSV inhibits the monocyte-endothelial cell adhesion and foam cell formation, which are likely to be partly responsible for suppression of atherogenesis produced by ligature-induced periodontitis.

### RSV inhibited the TNF-α-induced phenotypic changes of endothelial cells *in vitro*

**Figure 6:**
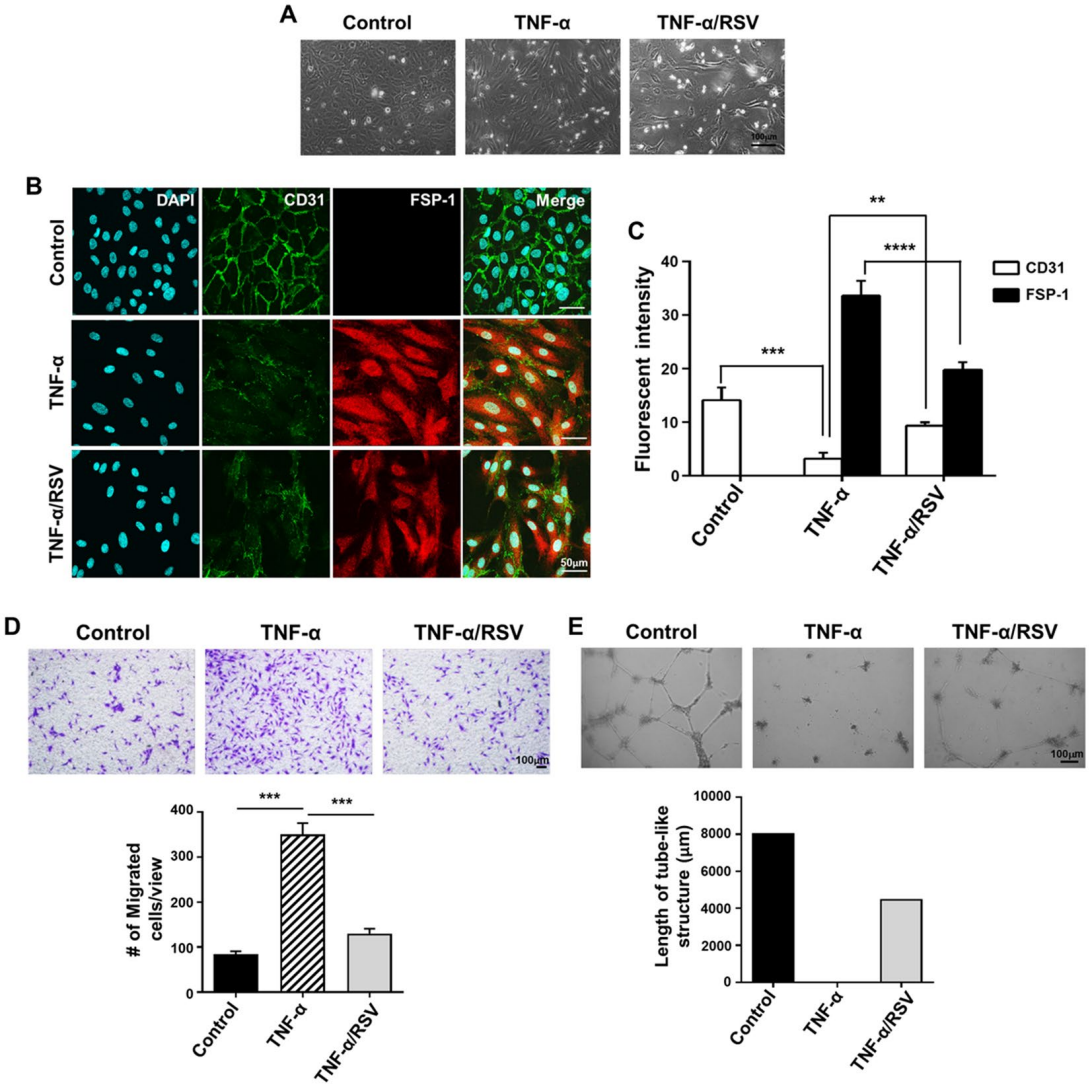
RSV partially abrogated TNF-α-induced phenotypic changes of HUVECs. (**A**) Phase contrast microscopy of HUVECs grown in basal media or media containing 50 ng/ml TNF-α with/without 10 μM RSV. (**B**) Immunofluorescence staining of HUVECs with anti-CD31 (Green) and anti-FSP-1 (Red) antibodies. (**C**) Quantification of CD31 or FSP-1 positive cells. Nuclei were counterstained with DAPI (Blue). (**D**) Mobility of HUVECs after treatment by using Transwell chamber with 8-μm pore size membrane and quantification of migrated cells. (**E**) Representative images of tube formation assay in Matrigel. Quantitative analysis of total length of vessel-like structures. Three replicated wells were set up for each group and five randomly selected views from each well were analyzed. **P < 0.01, ***P < 0.001 and ****P < 0.0001. Results represent the means ± SEM performed in triplicate.

The effect of TNF-α on endothelial-mesenchymal transition (EndMT) of HUVECs was investigated since the EndMT and the loss of endothelial barrier function are critical steps of atherogenesis^26^. As shown in Fig. 6A,B, TNF-α altered the morphology of endothelial cells to mesenchymal phenotypes, while suppressing the expression of cluster differentiation 31 (CD31) and enhancing the expression of fibroblast specific protein (FSP-1). RSV suppressed the TNF-α-induced mesenchymal transition and the upregulation of FSP-1, although the CD31 downregulation was not notably reversed by RSV (Fig. 6A–C). The transwell migration assay also confirmed enhanced migration of HUVECs by TNF-α; and RSV almost completely inhibited this effect of TNF-α (Fig. 6D). Furthermore, HUVECs’ innate function of forming tube-like structures is diminished by TNF-α, and this diminution was partially rescued by RSV (Fig. 6E). These data underscore the protective effect of RSV in endothelial cells from atherogenesis.

### RSV inhibited TNF-α-induced enhanced migration and proliferation of human coronary artery smooth muscle cells (HCASMCs)

**Figure 7:**
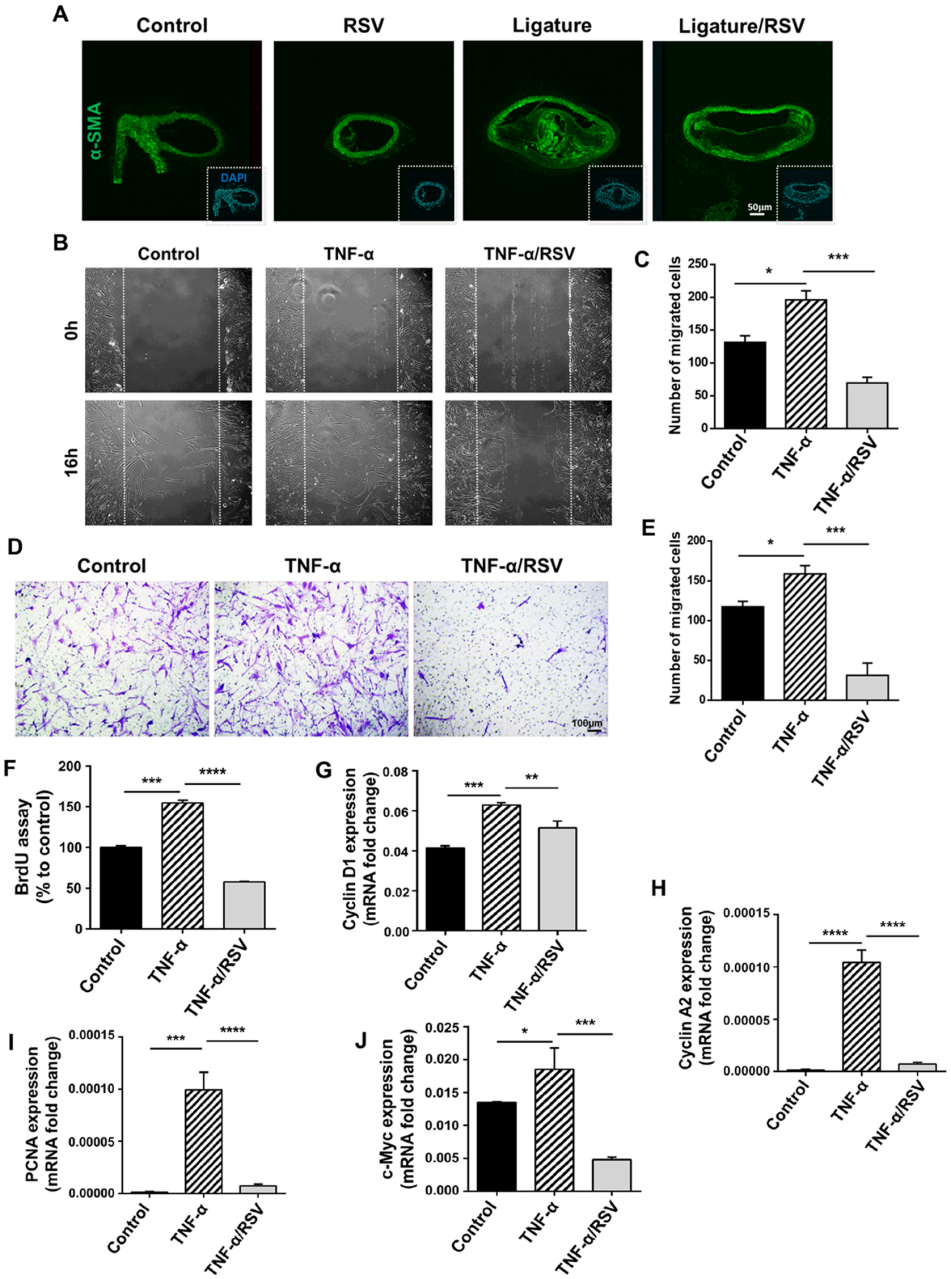
RSV inhibited the ligature-induced accumulation of smooth muscle cells (α-SMA positive) in the arterial plaques of mice. (A) *In vitro*, RSV suppressed the migration/ proliferation of human coronary artery smooth muscle cells (HCASMCs). Immunofluorescence staining of arterial plaques in brachiocephalic arteries with α-SMA antibody. Nuclei were counterstained with DAPI (Blue). (**B**) Migration/proliferation of the HCASMCs exposed to 50 ng/ml TNF-α with/without 10 μM RSV after scratch. The white dotted lines are margins of the scratches. (**C**) Quantification of cells in the scratched area. (**D**) Migration of HCASMCs exposed to 50 ng/ml TNF-α with/without 10 μM RSV through Transwell chamber with 8-μm pore size membrane. (**E**) Quantification of migrated cells. (**F**) BrdU assay demonstrating the cell proliferation in HCASMCs exposed to TNF-α and RSV. (**G**) Measurement of Cyclin D1 mRNA expression change in HCASMCs after TNF-α and RSV exposure. (**H**) Quantification of relative Cyclin A2 expression in control HCASMCs and those exposed to TNF-α and RSV. (**I**) Measurement of relative PCNA mRNA expression level post TNF-α and RSV treatment in HCASMCs. (**J**) Quantification of c-Myc mRNA expression level after TNF-α and RSV treatment in HCASMCs. *P < 0.05, **P < 0.01, ***P < 0.001 and ****P < 0.0001. Results represent the means ± SEM performed in triplicate.

VSMCs play important roles in atherogenesis and are implicated in the specific pathogenesis including migration, proliferation and morphologic changes, resulting in an accumulation of VSMCs in atherosclerotic plaques^27^. In our *in vivo* studies, we noticed that atherosclerotic plaques in mice with the ligature placement contained an abundance of cells expressing alpha smooth muscle actin (α-SMA), indicating high accumulation of cells with mesenchymal nature, such as VSMCs, in the plaques. This accumulation of mesenchymal cells was notably diminished by RSV administration (Fig. 7A), indicating possible blocking of inflammation-associated phenotypic alterations of VSMCs by RSV. TNF-α, the major inflammatory cytokine released from macrophages, is known to alter the phenotypes of VSMCs for atherogenesis^28^. To investigate the effect of RSV on such activity of TNF-α, we conducted *in vitro* studies using human coronary artery smooth muscle cells (HCASMCs). Exposure of HCASMCs to TNF-α induced enhanced migration of the HCASMCs, and RSV suppressed such effect of TNF-α (Fig. 7B–E). Furthermore, TNF-α increased the proliferation of HCASMCs, which was suppressed by RSV (Fig. 7F). Expression of the cell proliferation markers from HCASMCs such as Cyclin D1, Cyclin A2, proliferating cell nuclear antigen (PCNA) and cellular Myc (c-Myc) was significantly increased by TNF-α, and RSV abolished such effect of TNF-α (Fig. 7G–J). These data indicate that RSV possibly inhibits the development of atherosclerosis by, in part, suppressing the phenotypic changes of VSMCs induced by vascular inflammation.

### Ligature-induced periodontitis increased the level of cluster of differentiation 47 (CD47) in atherosclerotic plaques, and RSV diminished it by blocking the gene expression in HCASMCs

**Figure 8:**
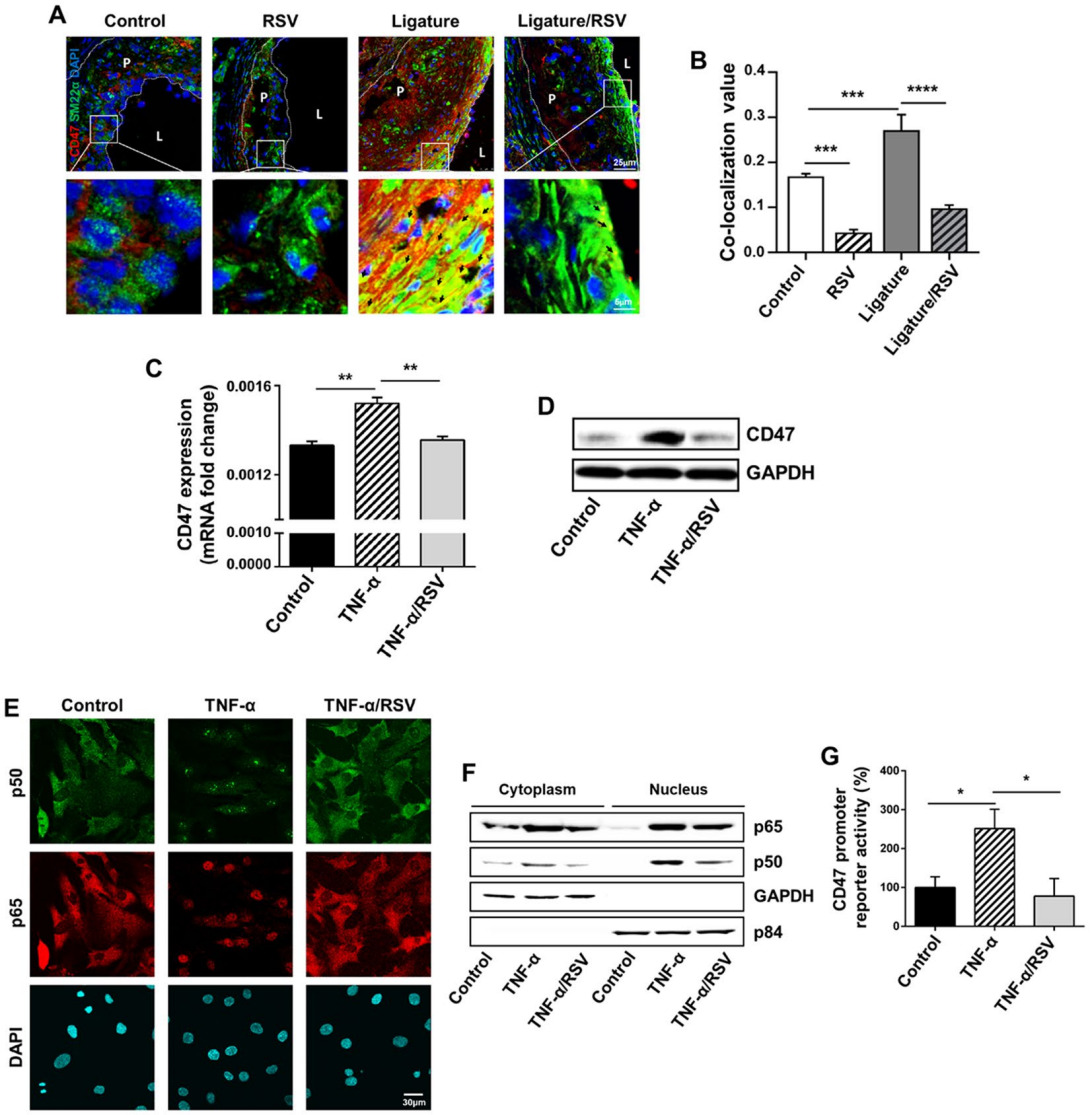
Abundant presence of CD47 in ligature-induced arterial plaques in mice, which was suppressed by RSV. Suppression of TNF-α-induced expression of CD47 in HCASMCs by RSV. (**A**) Immunofluorescence staining of atherosclerotic plaques in aortic roots with CD47 (Red) and SM22α (Green) antibodies. Nuclei were counterstained with DAPI (Blue). (**B**) Co-localization analysis of CD47 and SM22α based on the immunofluorescence staining images (Fig. 8A). (**C**) Relative expression level of CD47 in HCASMCs, TNF-α and TNF-α/RSV cell groups. (**D**) Western blot analysis of CD47 in HCASMCs, TNF-α and TNF-α/RSV cell groups. (**E**) Confocal immunofluorescence staining showing the activation status of NF-kB (p50 and p65) and nuclear protein stained with anti-DAPI. (**F**) Western blot demonstrating cytoplasmic and nucleic localization of NF-kB (p50 and p65). (**G**) Dual luciferase reporter assays for CD47 promoter activity of HCASMCs stimulated with TNF-α. *P < 0.05, **P < 0.01, ***P < 0.001 and ****P < 0.0001. Results represent the means ± SEM performed in triplicate.

To further examine the underlying molecular mechanisms, we performed microarray analysis on HCASMCs treated with or without TNF-α and found a significant overlap between cancer-related genes and cardiovascular disease-related genes including Cyclin A2, Cyclin D1, c-Myc, and PCNA, which were all significantly induced but negated by RSV treatment (Supplementary Fig. S6). Among them, CD47 is an essential key anti-phagocytic molecule in carcinogenesis and is known to prevent the elimination of cancer cells by macrophages^29,30^. As the advanced stage of atherosclerosis is characterized by the accumulation of vascular smooth muscle cells in the atherosclerotic necrotic core due to impaired clearance of these cells^31^, we determined the level of CD47 in the aortic root plaques. As shown in Fig. 8A,B, a moderate amount of CD47 was detected in the plaques of the control mice, and RSV notably reduced the level of CD47. In the aortic plaques of the mice with the ligature placement, we observed enhanced level of CD47, which was clearly reversed by RSV administration. To understand the mechanisms of RSV-induced inhibition of CD47 expression, we conducted *in vitro* studies using both mouse aortic smooth muscle cells (mASMCs) and HCASMCs exposed to TNF-α in the absence and presence of RSV. TNF-α remarkably enhanced the intracellular level and the expression of CD47, which was markedly suppressed by RSV in both mASMCs (see Supplementary Fig. S7) and HCASMCs (Fig. 8C,D). In addition, TNF-α exposure increased nuclear translocation of subunits (p50 and p65) of NF-κB, master inflammatory transcription factor, into nuclei of HCASMCs. RSV prevented the nuclear localization of NF-κB subunits induced by TNF-α (Fig. 8E,F). To determine whether RSV mechanistically regulates the transcription of CD47 gene expression induced by TNF-α, we performed the promoter reporter assay as previously described^32^. TNF-α treatment significantly enhanced the CD47 promoter activity, but this increase was drastically prevented by RSV (Fig. 8G). Collectively, these data indicate that effects of RSV on CD47 expression may partly account for prevention of atherosclerosis by RSV *in vivo*.

## Discussion

We previously established a periodontitis-induced atherosclerosis mouse model and demonstrated that periodontal disease leads to marked increase in atherosclerosis by inducing systemic and vascular inflammation^16^. In the current study, we investigated the therapeutic benefit of RSV in periodontitis-induced atherosclerosis. Our data revealed that RSV (1) reduced local inflammation and inhibited ligature-induced periodontal bone loss; (2) fully negated systemic inflammation caused by periodontitis; and (3) reduced exacerbation of atherosclerosis induced by periodontitis.

Statins are HMG-CoA reductase inhibitors that inhibit the mevalonate pathway. Although different statins, e.g., atorvastatin, lovastatin, pravastatin, simvastatin, or RSV have differential metabolic effects^33^, they can all unequivocally and effectively lower the LDL-C levels, which are the primary mechanisms by which statins reduce the risk of atherosclerotic cardiovascular diseases^7^. Accordingly, a number of clinical studies have demonstrated that statins are effective in preventing the risk of developing atherosclerosis^34^. In line with this notion, preclinical studies have demonstrated that statins can lower LDL-C levels and reduce atherosclerosis events in laboratory-induced atherosclerosis mouse models^35–38^, supporting the efficacy of statins in preventing atherosclerosis at both clinical and preclinical levels.

Despite numerous efforts to understand the preventive effects of statins on atherosclerosis, the role of statins on preventing periodontal diseases is relatively unknown. Previous studies have demonstrated the rescue effect of statins on periodontal diseases in the animals, although the degree of the reduction was marginally significant^39,40^. On the other hand, our study showed that RSV, one of the most potent statins, alone can significantly reduce the degree of periodontal diseases in mice; RSV treatment inhibited the alveolar bone loss and completely rescued the systemic inflammation mediated by ligature-induced periodontitis (Fig. 1; see Supplementary Fig. S1). This difference might be due to the different conditions of the experiment, such as different strains of mice (e.g., wild type vs. *ApoE*^−/−^ mice).

Consistent with our previous findings^16^, the ligature placement sufficiently induced local and systemic inflammation in mice, but those inductions were significantly negated by RSV (Figs. 1G and 2B). Although the primary mode of action for statins is to lower LDL-C level, increasing lines of evidence support the notion that statins also have lipid-independent pleiotropic effects including anti-inflammatory functions^41,42^. It was also proposed that the effects of statins on periodontal therapy did not depend on its lipid-lowering effects^43^. Indeed, our results revealed that RSV showed a direct role in preventing the differentiation of osteoclasts. As such, it is more plausible to speculate that the effect of RSV on periodontal disease is linked more to its anti-inflammatory functions rather than its lipid-lowering functions.

In spite of its strong anti-inflammatory effect, RSV failed to completely prevent the ligature-induced bone loss (Fig. 1). Given that the ligature placement around the tooth induces continuous mechanical tissue irritation in addition to the induction of inflammatory signaling, it is conceivable that continual source of mechanical irritation led to bone destruction even with RSV. In sharp contrast, the exacerbated atherosclerosis was significantly negated by RSV when the ligature was left in place (Fig. 3). Similarly, systemic and local inflammation around the atherosclerotic lesions was significantly reduced by RSV despite the presence of ligature (Figs. 2B and 4B). These data suggested that, even in the presence of periodontal diseases, reducing the systemic inflammation may help to prevent atherosclerosis development. It will be interesting to examine whether complete suppression of systemic inflammation by other means in the presence of ligature-induced periodontitis can notably prevent the exacerbation of atherosclerosis in mice.

It is noteworthy that, although RSV reduced periodontitis-induced atherosclerosis in both male and female mice, it did not confer any effects on the levels of TG, TC, LDL-C, and HDL-C in female mice. C57BL/6 is the most atherosclerosis-susceptible mouse stain with high variability in male and female^44^. Depending on different types of high-fat diet, *ApoE*^−/−^ mice exhibited different cholesterol levels^45^. A previous study showed that estrogen can also rescue atherosclerosis development without altering the plasma lipoprotein levels in *ApoE*^−/−^ mice^46^. These observations suggest that, at least in the context of *ApoE*^−/−^ mice in C57BL/6 background, the level of cholesterols does not correlate with atherosclerosis development.

At the local levels where atherosclerotic lesions were found, expression of TNF-α, IL-1β, and IL-6 were all suppressed significantly by RSV (Fig. 4B). Also, we found that macrophages are abnormally present in the atherosclerotic sites (Fig. 4A and see Supplementary Fig. S4), and macrophage adhesion and the expression of adhesion molecules were all increased by TNF-α (Fig. 5A–5D) and reversed by RSV on HUVECs. Advanced stage of atherosclerosis is characterized by the accumulation of diseased macrophages and vascular smooth muscle cells in the atherosclerotic necrotic core. Impaired clearance of these diseased macrophages and smooth muscle cells may potentiate and exacerbate vascular inflammation^31^. Furthermore, VCAM and ICAM are two surface molecules that are present on the endothelial cells and mediate macrophage-endothelial cell attachment, and VCAM, but not ICAM, has been shown to play a functional role in developing atherosclerosis^47^. These results highlight the direct involvement of RSV on negating the atherosclerosis development *via* lowering inflammatory signals.

Atherosclerosis and cancer are both chronic diseases in origin and share many molecular pathways including pro-inflammatory signals such as TNF-α^48^. In cancer biology, CD47 is an essential key anti-phagocytic molecule that provides “Don’t eat me” signals to evade the immune system for clearance^49^. A recent study demonstrated a determinant role of CD47 in atherosclerosis by mediating clearance of diseased macrophages and vascular smooth muscle cells^32^. In line with these notions, we found that RSV directly suppressed the TNF-α-mediated expression of CD47 (Fig. 8). Mechanistically, we found that it is associated with suppressing the localization of p50, one of the core NF-κB transcription factors, in the nucleus (Fig. 8E,F). Therefore, the preventive effects of RSV seem highly diverse and pleiotropic.

Similar to other animal models for recapitulating human diseases, the current study has several limitations. First, there are many different ways to induce periodontitis in animals^50^, and interpretation of our study should be carefully evaluated. Second, we used 12 weeks of ligature placement to induce periodontitis; however, this duration may not be applicable in other mouse periodontitis models^51^. Third, ligature placement is known to induce bone loss due to the accumulation of dental plaque and microulceration of the sulcular epithelium^50^; however, this is not the case in the clinical settings in which microbial infection components is the major pathological cause. Therefore, further studies are needed to evaluate the observation of the current study in the presence of periodontal bacterial infection alone.

A recent systemic review with meta-analysis showed that, based on 15 clinical studies examined, statin use as a sole adjunct to mechanical periodontal treatment improved the clinical periodontal parameters^52^. Similarly, recent guidelines from American College of Cardiology/American Heart Association stated that statin is the first-line of treatment for primary prevention of Atherosclerotic Cardiovascular Disease (ASCVD) in patients with elevated LDL-C levels^53^. Nonetheless, the association between periodontitis and atherosclerosis remains elusive^54^. Although we provide convincing evidence that severe periodontal disease can exacerbate atherosclerosis development *via* systemic inflammation, which can be prevented and mitigated by RSV in laboratory animals, our study needs clinical validations in human subjects in the future.

## Materials and Methods

### Induction of Periodontitis in mice

A total of 40 male *ApoE*^−/−^ mice that were 7 weeks old with C57BL/6 background were purchased from Jackson Laboratory (Bar Harbor, ME; Cat #002052) and housed in the vivarium at University of California, Los Angeles (UCLA), Division of Laboratory Animal Medicine. To determine the effect of Rosuvastatin on Periodontitis-induced Atherosclerosis, all mice were fed with a high-fat diet (HFD) (#D12079B, Research Diets, New Brunswick, NJ) for 1 week. One week after starting the HFD, these mice were divided into four groups (10 mice per group): (1) the Control group that received only HFD but no RSV and ligature; (2) the RSV group that received HFD containing RSV (RSV, Cayman Chemical, Ann Arbor, MI; 0.005%w/v) as previously described^37^; (3) the Ligature group that received HFD and subgingival ligature with 6-0 silk suture at the upper second molars; and (4) the RSV/Ligature group that received both HFD containing RSV and ligature placement. Ligature was placed under general anesthesia using Ketamine/Xylazine (100 mg per kg and 5 mg per kg, respectively) as described previously^17^. To examine the gender differences, the same experiment was performed using 40 female *ApoE*^−/−^ mice. All procedures were performed in compliance with the institution’s policy and applicable provisions of the United States Department of Agriculture (USDA) Animal Welfare Act Regulations and the Public Health Service (PHS) Policy. The experimental protocols were approved by the Animal Research Committee (ARC) of the University of California, Los Angeles (UCLA).

### Tissue collection

At the end of 14 weeks from the beginning of HFD feeding, we collected whole blood by cardiac puncture under general anesthesia with isoflurane (Abbott Laboratories, Lake Bluff, IL). The mice were then perfused and fixed with 4% paraformaldehyde in phosphate-buffered saline (PBS) *via* the left ventricle for 5 min. After the perfusion, the heart, the carotid artery and the full-length of the aorta-to-iliac bifurcation were exposed and dissected carefully from any surrounding tissues. The heart and artery samples were embedded in Optimal cutting temperature (OCT) compound (Fisher Scientific, Hampton, NH), and sectioned at 10 μm thickness. Twelve sections at 100 μm intervals were collected from each mouse for quantifying the lipid deposition with Oil red O staining and for additional immunohistochemical analysis on the sinus lesion. The maxillae were excised and the half of the palatal tissues were harvested using a blade, and the harvested palatal tissues were used to determine the expression of proinflammatory cytokines. For micro-computed tomography (μCT) analysis, the maxilla bones were fixed with 4% paraformaldehyde in PBS, pH 7.4, at 4 °C overnight and stored in 70% ethanol solution. After the fixation, decalcification continued for 3 weeks at 4 °C. The decalcification solution was changed daily. Decalcified maxillae and sectioned aorta were sent to the UCLA Translational Procurement Core Laboratory (TPCL) and processed for paraffin embedding. Blocks were sectioned at 5 μm intervals using a Microtome and slides were dewaxed in xylene.

### Micro-computed tomography (μCT) analysis

The fixed maxillae were subjected to μCT scanning (Skyscan1275, Bruker-microCT, Kontich, Belgium) using a voxel size of 20 μm^3^ and a 0.5 mm aluminum filter at 55 kVp and 145 μA. Two-dimensional slices from each maxilla were combined using NRecon and CTAn/CTVol programs (Bruker) to form a three-dimensional reconstruction. Bone loss was quantified by measuring the distance from the palatal and mesiobuccal cement-enamel junction (CEJ) to the alveolar bone crest (ABC).

### *En Face* analysis

The full-length of the aorta-to-iliac bifurcation was opened along the ventral midline and dissected free of the animal under a stereomicroscope (Stemi 305; Zeiss, Oberkochen, Germany). For *En face* analysis, the aorta was stained with Sudan IV (Sigma-Aldrich, St. Louis, MO) as previously described^55^ and pinned out flat, intimal side up, between cover slides. Aortic images were captured with a Nikon digital camera and analyzed using ImageJ software version 1.48 (NIH, Bethesda, MD; http://imagej.nih.gov/ij). To see accumulated lipid on aortic sinus, cross-sectioned artery and liver, sections were stained with Oil Red O. The percentage of lesion area was calculated as total lesion area divided by total surface area.

### Serum lipid and cytokine measurements

Levels of total cholesterol (TC), triglycerides (TG), and high density lipoprotein-cholesterol (HDL-C) were measured using enzymatic assay kits in the UCLA Cardiovascular Core Facility. VLDL-C (very low density lipoprotein-cholesterol) and LDL-C (low density lipoprotein-cholesterol) were calculated based on the Friedewald equation^56,57^. The serum level of TNF-α, IL-1β and IL-6 were measured by Enzyme-linked immunosorbent assay (ELISA) using Ready-SET-go kits (Thermo Fisher Scientific, Waltham, MA) according to manufacturer’s protocol. The color reaction was stopped with the addition of Stop solution (BioLegend, San Diego, CA), and absorbance was read immediately using a plate reader at 450 nm (Bio-Rad Laboratories, Hercules, CA). The standard curve was calculated by plotting the standards against the absorbance values, and the cytokine levels were measured in pg/ml.

### Histological and immunofluorescence analysis

For tartrate-resistant acid phosphatase (TRAP) staining, the sections were stained using an acid phosphatase kit (378 A; Sigma-Aldrich) and then counterstained with hematoxylin. The digital images of the histochemical stained section were obtained using the microscope (DP72, Olympus, Tokyo, Japan). Mouse OCT-embedded aortic sinus was cut and stained by Von Kossa for calcium salts and Masson’s trichrome staining for collagen deposition. For immunohistostaining, the sections were incubated with primary antibodies, MOMA-2 (Bio-Rad Laboratories), CD47 (Novus Biologicals, Centennial, CO) and SM22α (Abcam, Cambridge, United Kingdom), followed by fluorometric detection with Alexa Fluor 488- or Alexa Fluor 594-conjugated secondary antibodies (Thermo Fisher Scientific). Sequentially, the sections were mounted on slides with VECTASHIELD^TM^ anti-fade mounting medium with 4′,6-diamidino-2-phenylindole (DAPI) (H1200, Vector laboratories, Burlingame, CA). Slides were investigated with a Fluoview FV200i confocal fluorescent microscope (Olympus).

### *Ex vivo* osteoclastogenesis

To obtain bone marrow derived macrophages (BMDMs), bone marrow cells were isolated by flushing the femurs and tibias of 6–8 week-old C57BL/6 J mice (Jackson Laboratory). BMDMs were cultured in a minimum essential medium (Thermo Fisher Scientific) supplemented with 10% fetal bovine serum and 1% antibiotic-antimycotic solution and differentiated with 30 ng/ml macrophage-colony stimulating factor (M-CSF, R&D Systems, Minneapolis, MN, USA) for 3 days. To induce osteoclastogenesis, BMDMs were further cultured with M-CSF and 100 ng/ml receptor activator of nuclear factor kappa-B ligand (RANKL, R&D Systems) with or without 10 μM RSV for 0–3 days. After 0–3 days, cells were fixed and stained for TRAP activity by using the acid phosphatase kit (378 A; Sigma-Aldrich) according to manufacturer’s instructions or harvested for quantitative RT-PCR (qRT-PCR).

### Cell culture and reagents

Human umbilical vein endothelial cells (HUVECs; Lonza, Basel, Switzerland) were cultured in endothelial basal medium-2 containing EGM-2 SingleQuot Kit (Lonza). Human coronary artery smooth muscle cells (HCASMCs; Lonza) were cultured in SmBM smooth muscle cell growth basal medium containing SmGM-2 SingleQuot Kit (Lonza). The human monocytic leukemia cell line (THP-1) was purchased from ATCC (Manassas, VA) and cultured in RPMI1640 medium containing 10% fetal bovine serum (FBS, Thermo Fisher Scientific) and 1% penicillin/streptomycin. The medium was renewed every 48 h. Cells were cultured at 37 °C and in CO_2_ air atmosphere with a humidity of 5% (v/v). 100 nM Phorbol 12-myristate 13-acetate (PMA; Sigma-Aldrich) was treated to differentiate THP-1 cells into macrophages, and untreated THP-1 cells were used as monocytes.

Mouse aortic smooth muscle cells (mASMCs) were isolated from thoracic aortas of 6-week-old C57BL/6 mice. Briefly, perivascular fat-free aortas were harvested, and were digested with collagenase I (1 mg/ml, Thermo Fisher Scientific), elastase (0.744 U/ml, Worthington Biochemical Corp., Lakewood, NJ) Soybean Trypsin inhibitor (1 mg/ml, Worthington Biochemical Corp.) in HBSS (Thermo Fisher Scientific). After 8 min digestion, the adventitia was removed, and the endothelial cell layer was removed from the opened aorta. For mASMC isolation, aortas were cut into ~0.5-mm pieces and placed in the above enzyme cocktail for an hour to complete the digestion. The mASMCs were then grown and maintained in DMEM/F12 medium with 20% fetal bovine serum (FBS, Thermo Fisher Scientific) and 1% antibiotic-antimycotic (Thermo Fisher Scientific).

### Cell migration assay

Twenty-four transwell chambers were used to monitor tumor cell migration and invasion. Briefly, cells were pre-treated with 10 μM RSV for 3 h and then transferred to a transwell chamber with 8-μm pore size membrane in the medium with 0.5% FBS. The growth medium containing 10% FBS with 50 ng/ml TNF-α was placed in the lower chamber of the transwell chamber. After 13 h incubation, cells on the upper side of the membrane were removed by wiping with a cotton swab and stained with crystal violet, and then the migrated cells were micro-photographed.

### Tube formation assay

HUVECs (2 × 10^4^ cells/well) were seeded on a 96-well plate and cultured in serum-free basal media in the presence of 10 μM RSV or PBS (control) for 3 h and then treated 50 ng/ml TNF-α for 1 day. The cells were trypsinized and placed onto the Matrigel (BD Biosciences, San Jose, CA)-coated wells of a 96-well plate in complete media with TNF-α. Tube formation was examined using a phase-contrast microscope (Olympus, Tokyo, Japan) and the total length of the network was evaluated in five randomly selected fields for each well. The total length of the network was measured using ImageJ.

### *In vitro* migration assay

The HCASMCs were seeded on 12-well plates at a concentration of 6×10^5^ cells/ml cultured in the medium containing 0.5% FBS and grown to attach cells for 24 h at 37 °C and 5% CO_2_ and the cells were pre-treated 10 μM RSV or PBS (control) for 3 h. Subsequently, a small area was scratched using a sterile 200 μl pipette tip before TNF-α treatment. The cells were extensively rinsed with PBS to remove the loosened debris of the cells. After 16 h of TNF-α treatment at 37 °C, the distance between two layers of cells, which were scratched by a pipette tip, was then inspected microscopically. For observation of the wound area closure, the migration of cells after filling scratched area, was captured by a digital camera attached to an inverted microscope and computer system.

### Quantitative real-time polymerase chain reaction

Total RNA from mouse tissues or human/mouse cells was extracted using RNeasy micro kit (Qiagen, Hilden, Germany) and reverse-transcribed using SuperScript® III Reverse Transcriptase Synthesis Kit (Thermo Fisher Scientific). Subsequently, qRT-PCR was performed using PowerUp™ SYBR Green Master Mix (Thermo Fisher Scientific) or TaqMan primers (Applied Biosystems, Foster City, CA) according to manufacturer’s protocol. The sequences of the primers used for qRT-PCR were described in Table in Online Supplementary Data. Glyceraldehyde 3-phosphate dehydrogenase (GAPDH) served as control and the fold induction was calculated using the comparative ΔCt method and presented as relative transcript levels (2^−ΔΔCt^).

### Immunoblotting

Total protein was extracted and size-fractioned by SDS-polyacrylamide gel electrophoresis and transferred to nitrocellulose membranes. After blocking with 5% skim milk in PBS with 0.1% Triton-X100, immunodetection was carried out using specific primary antibodies: anti-VCAM (Abcam), anti-ICAM (BioLegend), anti-p50 (Santa Cruz Biotechnology), anti-p84 (Santa Cruz Biotechnology), anti-p65 (Santa Cruz Biotechnology, Dallas, TX), anti-FSP-1 (Abcam), anti-CD31 (Abcam), anti-VE-Cadherin (Abcam), anti-α-SMA (Sigma-Aldrich).Glyceraldehyde 3-phosphate dehydrogenase (GAPDH) (Santa Cruz Biotechnology); was used as loading control. Thereafter, blots were incubated with HRP-labeled respective (anti-mouse or anti-rabbit) secondary antibodies (Santa Cruz Biotechnology), washed and processed with Clarity™ Western ECL Substrate (Thermo Fisher Scientific). Signal was detected using ChemiDoc™ Touch image analyzer (Bio-Rad Laboratories).

### Immunocytochemistry

Immunostaining was performed in 4-well chamber slide. After fixing with 4% paraformaldehyde for 10 min and washing with PBS, blocking solution (5% bovine serum albumin in PBS with 0.1% Triton-X100) was applied for 1 h. Primary antibodies (anti-CD31: Abcam, anti-FSP-1: Abcam, anti-VE-Cadherin: Abcam and anti-α-SMA: Sigma-Aldrich) were applied overnight at 4 °C. Cells were washed with PBS, and corresponding fluorescence-tagged secondary antibodies were applied for 1 h at room temperature. After washing, the cells were mounted using VECTASHIELD^TM^ anti-fade mounting medium with DAPI (Vector laboratories). Immunostaining was observed under Olympus Fluoview FV200i confocal fluorescent microscope (Olympus).

### Enzyme‐linked immunosorbent assay (ELISA)

Levels of IL-6, IL-1β and TNF-α in supernatants from THP-1 derived cells were measured by ELISA using Ready-SET-go kits (Thermo Fisher Scientific) according to manufacturer’s protocol. The color reaction was stopped with the addition of Stop solution (BioLegend), and absorbance was read immediately using a plate reader at 450 nm (Bio-Rad Laboratories). The standard curve was calculated by plotting the standards against the absorbance values, and the cytokine levels were measured in pg/ml.

### Cell adhesion assay

After labeling with Calcein-AM (Sigma-Aldrich), THP-1 cells were co-cultured on monolayers of HUVECs in chamber slides, exposed to either culture medium only or TNF-α for 1 h. Thirty minutes after the co-culture, we washed the culture thoroughly and evaluated the attached THP-1 cells to HUVECs using Olympus Fluoview FV200i confocal fluorescence microscope (Olympus) as described elsewhere^16^.

### CD47 promoter activity assay

To construct CD47-luciferase vector, we cloned the promoter region of CD47 LightSwitch Promoter Reporter GoClones (RenSP, S710450; SwitchGear Genomics, Menlo Park, CA) to pGL3-basic vector (Promega, Madison, WI). For transfection reference, we used the reference vector containing Renilla luciferase region (Promega). Then HCASMCs were transfected with 45 ng of the CD47-luciferase vector and 5 ng of the reference vector using Lipofectamine 2000 (Invitrogen) for 48 h. At the end of transfection period, we washed the cells and cultured the cells in media containing 20 µM RSV for 3 h. After the removal of culture media containing RSV, we continuously cultured cells with or without 100 ng/ml of TNF-α for 24 h. Then the cell lysates were collected, and dual luciferase activity was measured with the Dual-Luciferase Reporter Assay System (Promega), according to the manufacturer’s instructions. Relative luciferase activity (Firefly/Renilla luciferase ratio) was quantified as the percentage change relative to the basal values obtained from control-transfected cells not exposed to TNF-α treatment.

### RNA sequencing and bioinformatics analysis

RNA quality assessment, high throughput sequencing services and bioinformatics analysis in TNF-α treated HCASMCs were conducted by UCLA Technology Center for Genomics & Bioinformatics (TCGB) at UCLA. Briefly, the cells were treated by TNF-α for 6 h and lysed using RLT Buffer (Qiagen) supplemented with 1% β-mercaptoethanol. Total RNA was then isolated using RNeasy Micro kit (Qiagen) according to manufacturer’s protocol and quantified by Nanodrop 1000 (Thermo Fisher Scientific). All samples had RNA integrity above 8.0 as determined by Bioanalyzer (Agilent Technologies). RNA library preparation was performed using Universal Plus mRNA Kit (Nugen, Redwood city, CA) as per manufacturer’s protocols. Final libraries were multiplexed samples in one lane and sequenced on a HiSeq. 3000 sequencer (Illumina, San Diego, CA) generating 50 bp single-end reads. Sequenced reads were demultiplexed with Bcl2fastq2 v 2.17 program (Illumina), mapped to the latest UCSC transcript set using Bowtie2 (version 2.1.0), and the gene expression level was estimated using RSEM v1.2.15. TMM (trimmed mean of M-values) was used to normalize the gene expression. Mapping statistics were exceptional; between 80.88% and 84.41% of reads mapped to the transcriptome for any sample. Genes were clustered with Cluster 3.0 using single linkage and Pearson correlation as similarity measure and heatmap was generated by TreeView. The ‘Disease and Functions’ analysis was performed using Ingenuity Pathway Analysis (IPA; Qiagen).

### Nuclear translocation of NF-κB signals

After plating 5 × 10^4^ cells per well of 4-well chamber slides, the cells were pretreated with 20 µM of RSV for 3 h. Then 100 ng/ml TNF-α was added to the culture for 20 min to activate the NF-κB translocation. Following the activation, cells were fixed for 10 min in 4% PFA and the primary polyclonal NF-κB/p65 and NF-κB/p50 antibodies (Santa Cruz Biotechnology) were diluted 1:200 in 5% BSA/PBS and added to PFA-fixed cells after blocking with 10% bovine serum albumin (BSA)/PBS for 30 min. Then, 1:1000 dilution of secondary fluorescence conjugated antibodies (Jackson ImmunoResearch Laboratories Inc., West Grove, PA) was added and incubated at room temperature in the dark for 50 min. Sequentially, the slides were mounted with VECTASHIELD^TM^ anti-fade mounting medium with DAPI (Vector laboratories). Slides were then examined with Fluoview FV200i confocal fluorescent microscope (Olympus).

### Statistical analyses

All graphs were created using GraphPad Prism software, and statistical analyses were calculated using GraphPad Prism 5. For multiple comparisons, 1-way ANOVA with Newman-Keuls test was used. A *P*-value of less than 0.05 was considered significant. All results from *in vitro* were confirmed by at least 3 independent experiments. Error bars represent mean ± SEM.

## Supplementary information

Supplementary information.
