# Supplementary material for: Rosuvastatin Prevents the Exacerbation of Atherosclerosis in Ligature-Induced Periodontal Disease Mouse Model
Source: Sci Rep. 2020 Apr 14;10:6383. doi: 10.1038/s41598-020-63350-8 (PMC7156392; doi:10.1038/s41598-020-63350-8)
Supplement: Supplementary file 1 — Supplementary information. [file 41598_2020_63350_MOESM1_ESM.pdf]

Supporting Information for

Rosuvastatin Prevents the Exacerbation of Atherosclerosis in Ligature-Induced Periodontal Disease Mouse Model

Jin Sook Suh[1], Sung Hee Lee[1], Zachary Fouladian[2], Jae Young Lee[1], Terresa Kim[1], Mo K. Kang[1], Aldons J. Lusis[2], Kristina I. Boström[2], Reuben H Kim[1, 3*], and No-Hee Park[1, 2, 3*]

[1]The Shapiro Family Laboratory of Viral Oncology and Aging Research, UCLA School of Dentistry, 10833 Le Conte Ave, Los Angeles, CA, USA

[2]Department of Medicine, David Geffen School of Medicine at UCLA, 10833 Le Conte Ave, Los Angeles, CA, USA

[3]UCLA Jonsson Comprehensive Cancer Center, 10833 Le Conte Ave, Los Angeles, CA, USA

Running title: Rosuvastatin averts Atherogenesis by Periodontitis

Correspondence:
*No-Hee Park, DMD, PhD
43-005 CHS, Box 951668, University of California, Los Angeles, CA 90095-1668
310-825-0339 (voice), nhpark@ucla.edu (email) or

*Reuben H. Kim, DDS, PhD
43-009 CHS, Box 951668, University of California, Los Angeles, CA 90095-1668
310-825-7312 (Voice), rkim@dentistry.ucla.edu (email)

Table of Contents

Supplementary Figures

Supplementary Figure S1. Osteoclast differentiation with and without RSV treatment in bone marrow cells isolated from WT and *ApoE*$^{-/-}$ mice
Supplementary Figure S2. RSV reduced the severity of periodontal, systemic and vascular inflammation induced by ligature placement in female *ApoE*$^{-/-}$ mice
Supplementary Figure S3. Expression levels of pro-inflammatory cytokines (TNF-α, IL-1β and IL-6) from spleen determined by qRT-PCR
Supplementary Figure S4. Ligature placement significantly increased collagen and calcium deposition on the aortic root, and RSV almost completely blocked Ligature-induced collagen and calcium deposition
Supplementary Figure S5. Atherosclerotic plaque accumulation status in mice
Supplementary Figure S6. Analysis of activated genes by TNF-α treatment in HCASMCs
Supplementary Figure S7. Expression level of CD47 gene following the inflammation on mASMCs *in vitro*
Supplementary Figure S8. Source data for Figures 5, 8, and Supplementary Figure S7
Supplementary Table S1. List of primers used in this study

Supplementary Fig. S1 Online: Osteoclast differentiation with and without RSV treatment in bone marrow cells isolated from WT and *ApoE*$^{-/-}$ mice

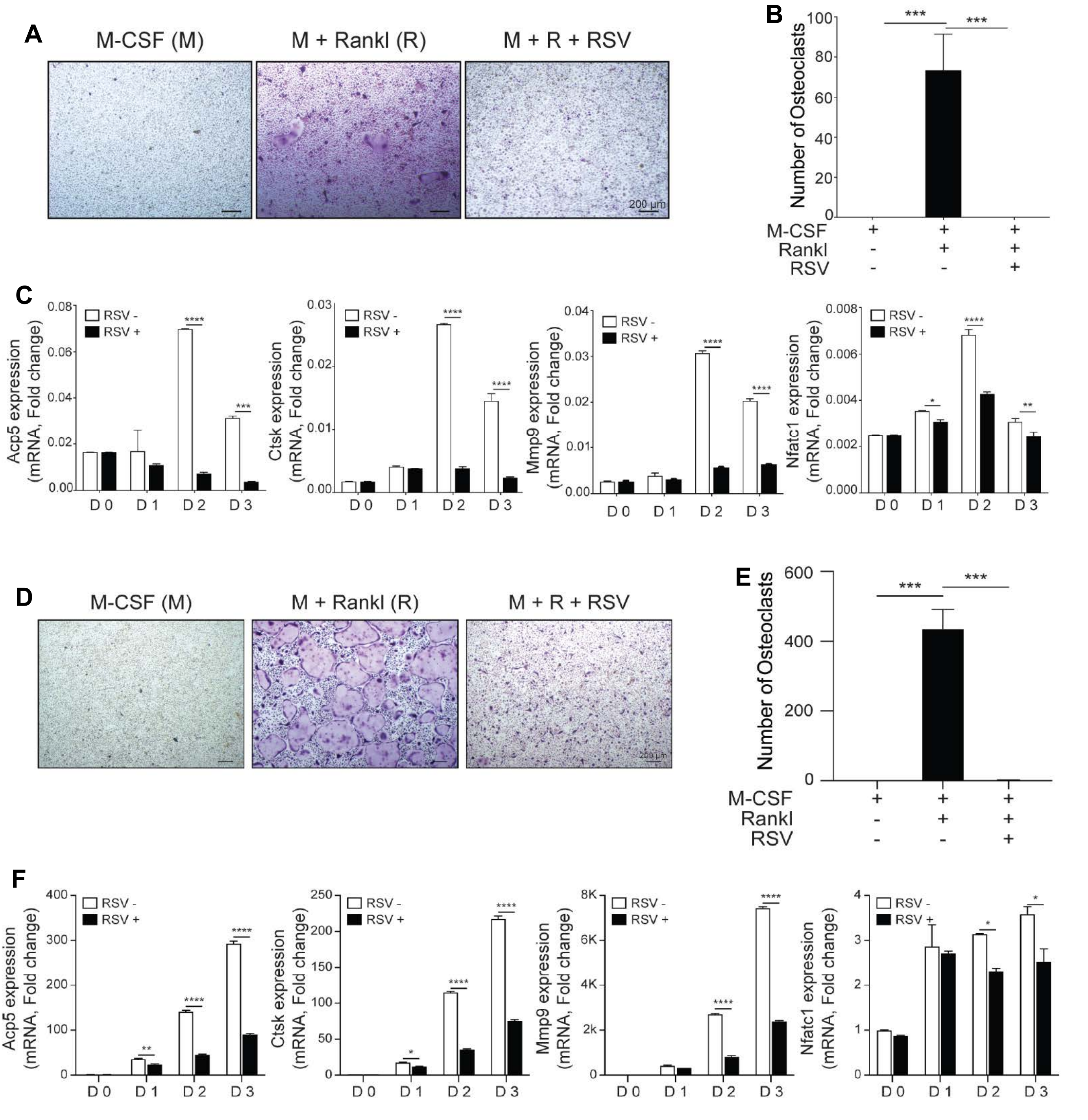

Supplementary Fig. S1. Osteoclast differentiation with and without RSV treatment in bone marrow cells isolated from WT or *ApoE*$^{-/-}$ mice. A-C, Osteoclastogenesis induced on BMMs from C57BL/6 mice. **A,D,** Development of osteoclasts was monitored by TRAP staining. Representative images of the cultures. **B,E,** Quantification of TRAP-positive multinucleated (>5 nuclei) osteoclasts per well in. **C,F,** Effect of RSV on the expression of the osteoclast marker genes (NFATC1, CtsK, ACP5, and MMP9) were measured by qRT-PCR. **D-F,** Osteoclastogenesis induced on BMMs from *ApoE*$^{-/-}$ mice. D0, Day 0; D1, Day 1; D2, Day 2; D3, Day 3 after incubation with RANKL. GAPDH served as loading control. $*P < 0.05$ and $****P < 0.0001$. Results represent the means ± SEM performed in triplicate.

Supplementary Fig. S2 Online: RSV reduced the severity of periodontal, systemic and vascular inflammation induced by ligature placement in female *ApoE*$^{-/-}$ mice

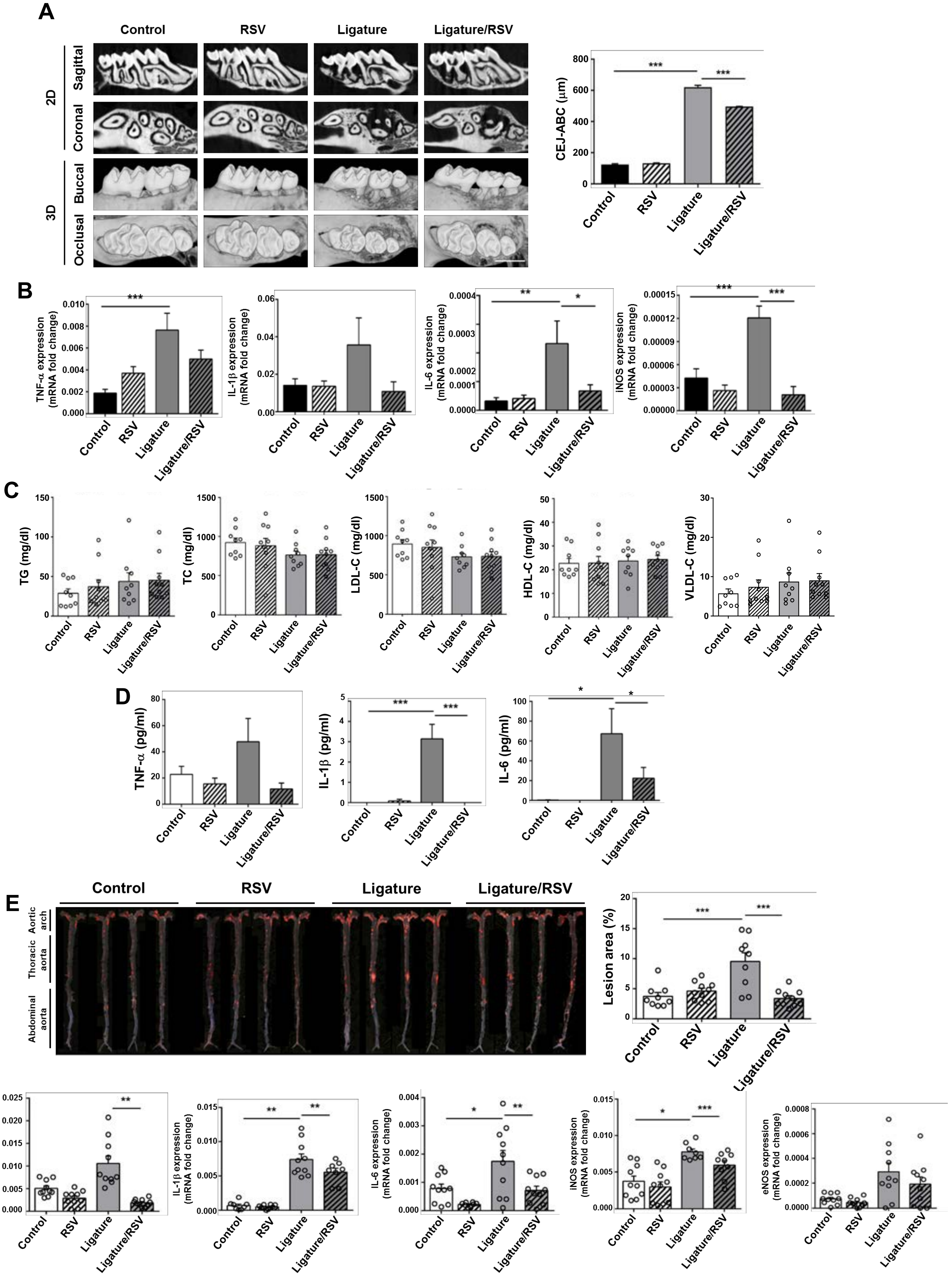

Supplementary Fig. S2. RSV reduced the severity of periodontal, systemic and vascular inflammation induced by ligature placement in female *ApoE*$^{-/-}$ mice. A, Representative two dimensional or three dimensional µCT images of mouse maxilla. Scale bar: 1 mm. Alveolar bone loss measured at the distobuccal (DB) root of the maxillary second molars from cemento-enamel junction (CEJ) to alveolar bone crest (ABC). **B,** Expression levels (determined by qRT-PCR) of pro-inflammatory cytokines (TNF-α, IL-1β, and IL-6) and iNOS at the palatal tissue of second molars. GAPDH served as loading control. **C,** Levels of Serum lipid; triglyceride (TG), total cholesterol (TC), non-high density lipoprotein cholesterol (Non-HDL-C) and high-density lipoprotein cholesterol (HDL-C). **D,** Levels of TNF-α, IL-1β and IL-6 from the mice sera by which were measured by using pre-coated ELISA plates. GAPDH served as loading control. **E,** Photographs and quantification of mice aortas from the *en face* preparation after staining with Sudan IV. **F,** Levels of gene expression (TNF-α, IL-1β, IL-6, iNOS and eNOS) from aortas determined by qRT-PCR. GAPDH served as loading control. $*P < 0.05$, $**P < 0.01$, and $***P < 0.001$. Results represent the means ± SEM performed in triplicate.

Supplementary Fig. S3 Online: Expression levels of pro-inflammatory cytokines (TNF-α, IL-1β and IL-6) from spleen determined by qRT-PCR.

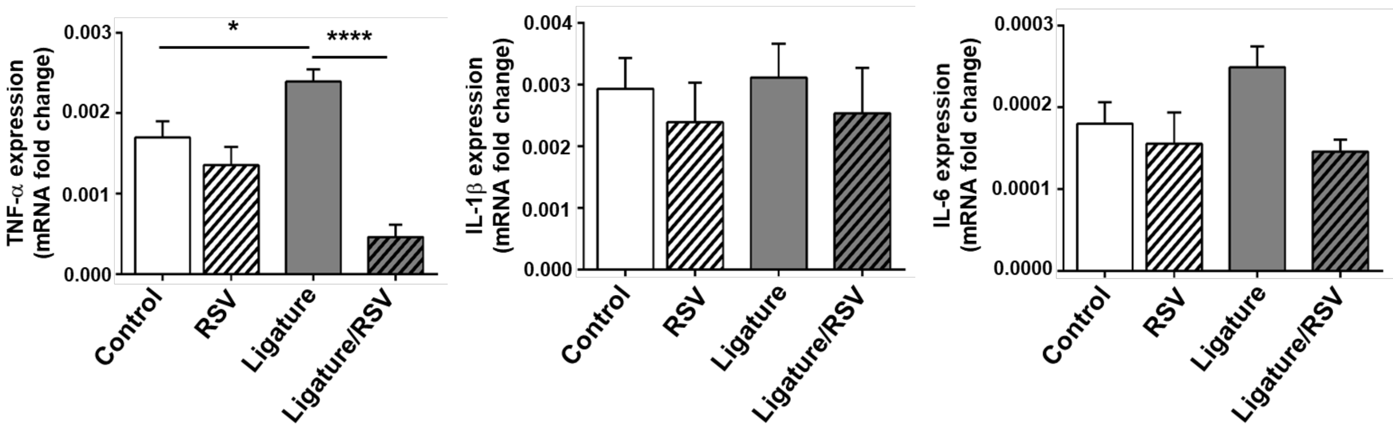

Supplementary Fig. S3. Expression levels of pro-inflammatory cytokines (TNF-α, IL-1β and IL-6) from spleen determined by qRT-PCR. GAPDH served as loading control. $*P < 0.05$ and $****P < 0.0001$. Results represent the means ± SEM performed in triplicate.

Supplementary Fig. S4 Online: Ligature placement significantly increased collagen and calcium deposition on the aortic root, and RSV almost completely blocked Ligature-induced collagen and calcium deposition

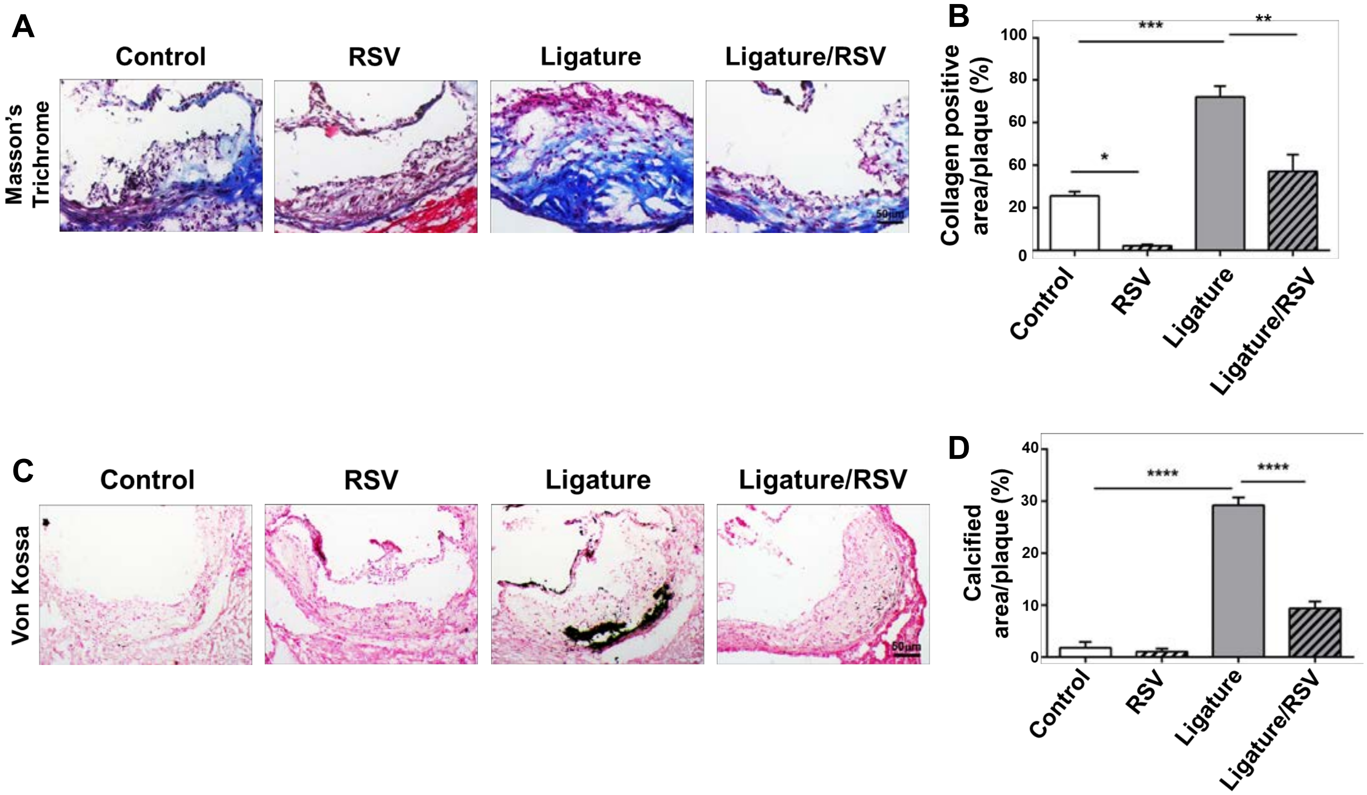

Supplementary Fig. S4. Ligature placement significantly increased collagen and calcium deposition on the aortic root, and RSV almost completely blocked Ligature-induced collagen and calcium deposition. A, Representative images of Masson's trichrome-stained aortic root stained as blue, which indicates collagen deposition. **B,** Quantification of Masson's trichrome-stained aortic root area. **C,** Representative images of Von Kossa-stained aortic root stained as black, which indicates calcium salt deposition. **D,** Quantification of the calcified lesion of the aortic root. GAPDH served as loading control. $*P < 0.05$, $**P < 0.01$, and $***P < 0.001$ and $****P < 0.0001$. Results represent the means ± SEM performed in triplicate.

Supplementary Fig. S5 Online: Atherosclerotic plaque accumulation status in mice.

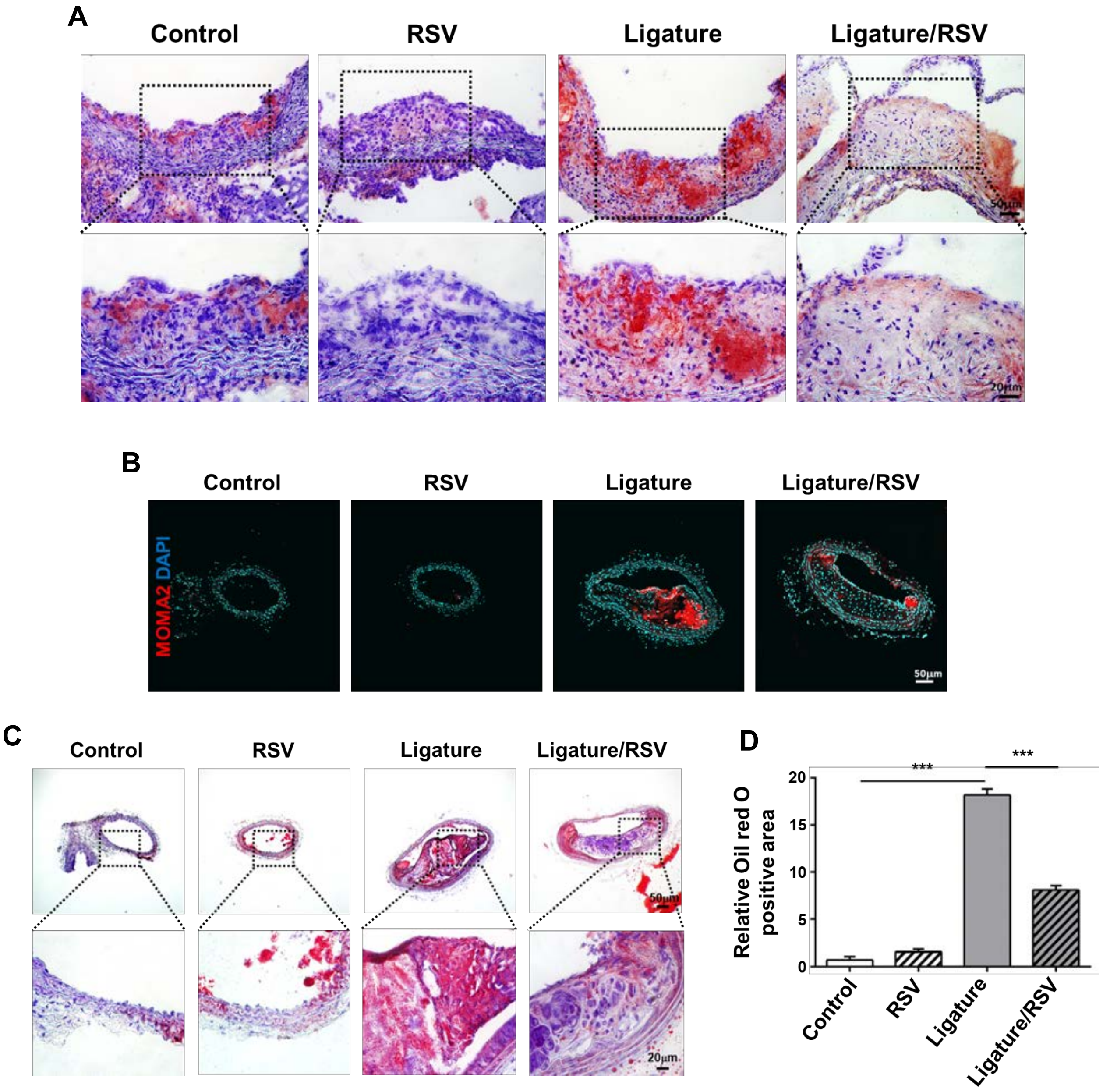

Supplementary Fig. S5. Atherosclerotic plaque accumulation status in mice. A, Histological analysis of atherosclerotic plaques in aortic roots with MOMA-2 antibody (Red). Nuclei were counterstained with Hematoxylin (Purple). **B,** Histological analysis of atherosclerotic plaques in brachiocephalic arteries with MOMA-2 antibody. Nuclei were counterstained with DAPI (Blue). **C,** Representative images of Oil Red O-stained brachiocephalic arteries from the mice. Nuclei were counterstained with Hematoxylin (Purple). **D,** Quantification of lesion area stained by Oil Red O.

Supplementary Fig. S6 Online: Analysis of activated genes by TNF-α treatment in HCASMCs

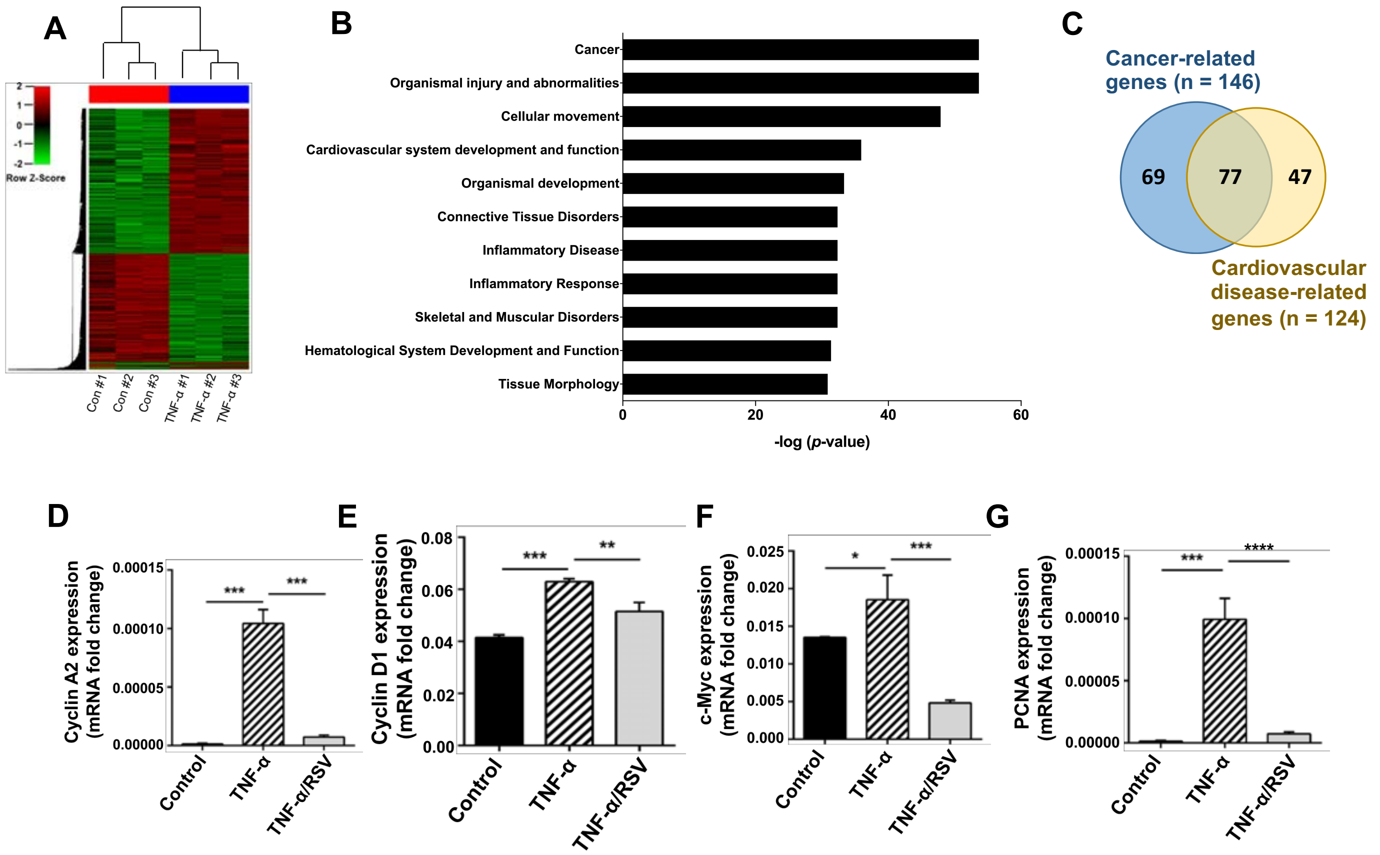

Supplementary Fig. S6. Analysis of activated genes by TNF-α treatment in HCASMCs. A, Heatmap of clustered genes induced by TNF-α (n = 3 per group). Color green indicates downregulated genes and color red indicates upregulated genes. **B,** IPA Disease and Functions analysis of TNF-α induced-gene signature. The *p*-value is calculated with the right-tailed Fisher's Exact Test and measures the significant overlap between the dataset genes and the genes that belong to the 'Disease and Function' categories in the IPA knowledge database. **C,** Venn diagram of cancer-related genes (n = 146) and cardiovascular disease-related genes (n = 124) identified 77 genes that are commonly expressed. Gene expressions of **D,** Cyclin A2, **E,** Cyclin D1, **F,** c-Myc and **G,** PCNA in TNF-α treated HCASMCs with/without RSV by using qRT-PCR analysis.

Supplementary Fig. S7 Online: Expression level of CD47 gene following the inflammation on mASMCs *in vitro*.

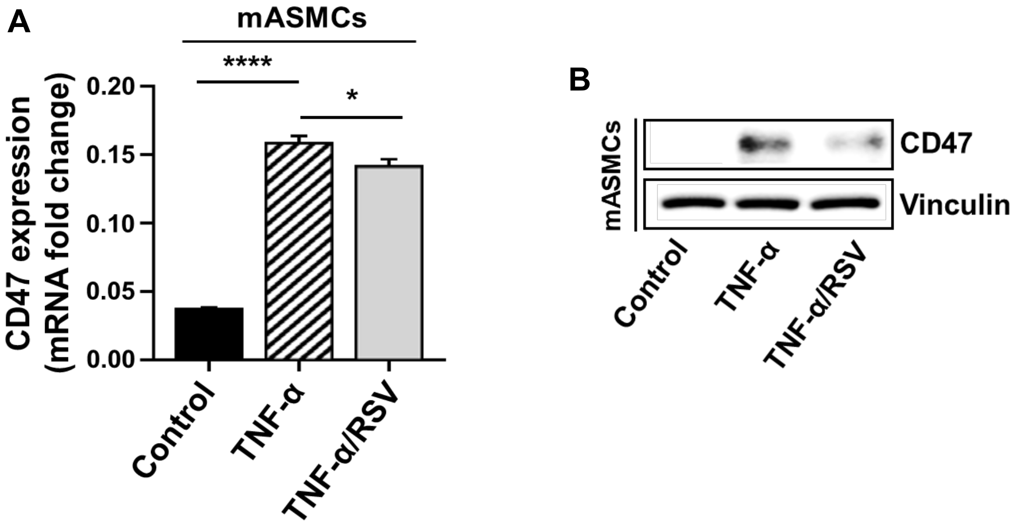

Supplementary Fig. S7. Expression level of CD47 gene following the inflammation on mASMCs *in vitro*. A, Relative CD47 mRNA expression in mASMCs and cells exposed to TNF-α alone or together with RSV. **B,** Protein expression level of CD47 in mASMCs alone or with TNF-α and TNF-α with RSV.

Supplementary Figure S8. Source data for Figures 5, 8, and Supplementary Figure S7

Source data for Figure 5: CD31, VE-Cad, VCAM, ICAM and GAPDH

CD31

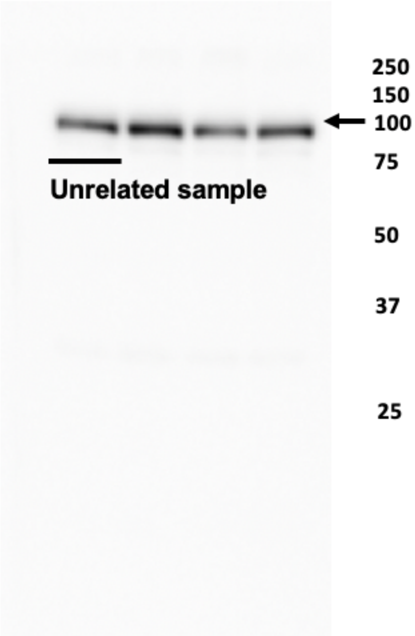

VE-Cad

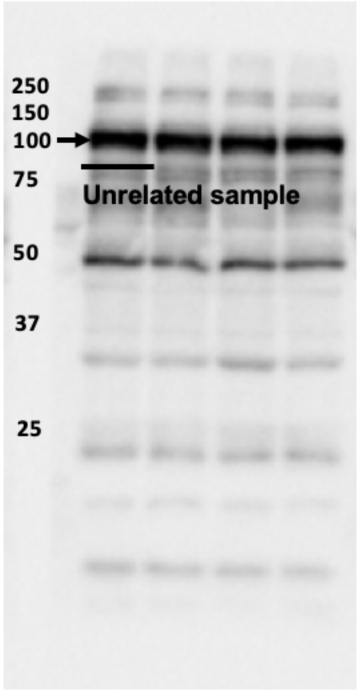

VCAM

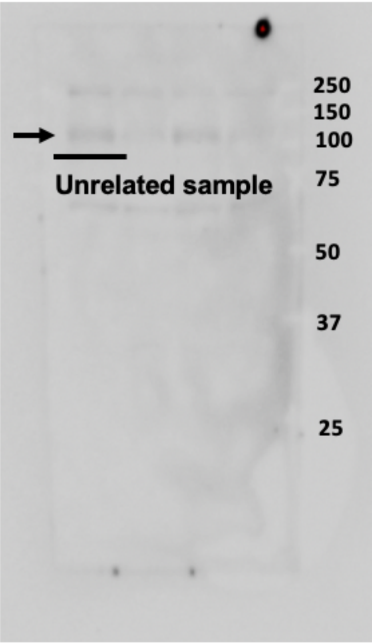

ICAM

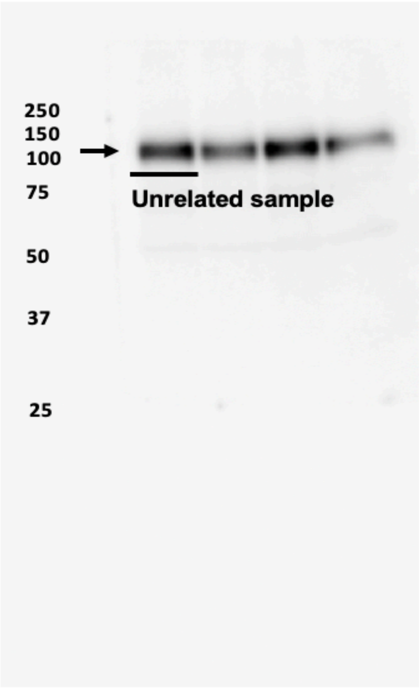

GAPDH

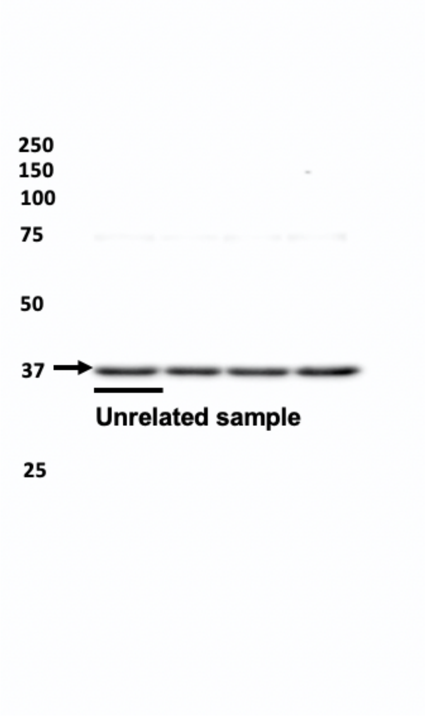

White light

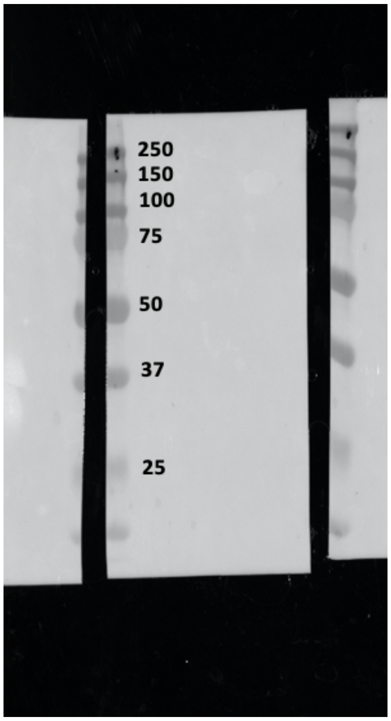

Source data for Figure 8D: CD47 and GAPDH

CD47

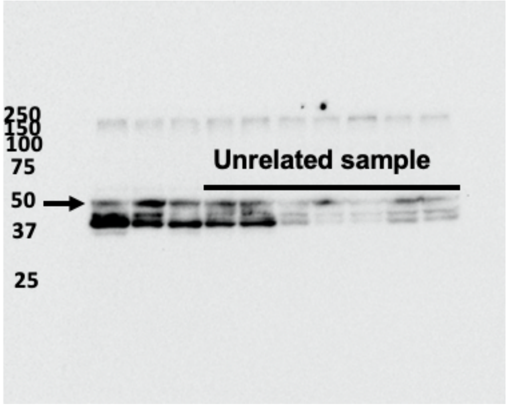

GAPDH

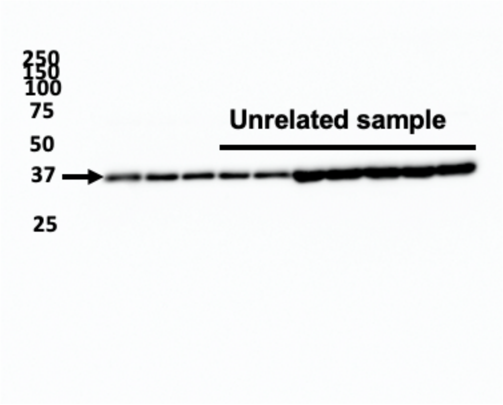

White light

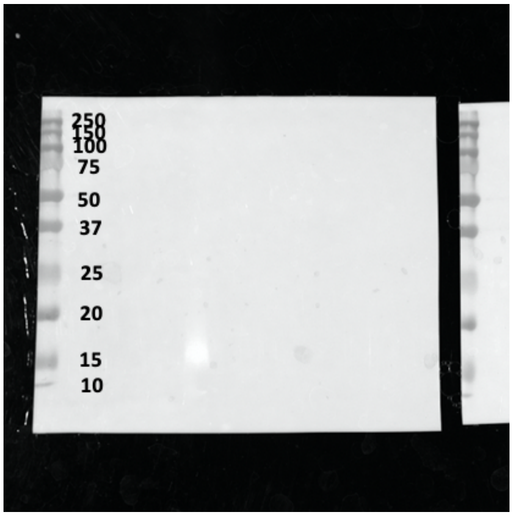

Source data for Figure 8F: p65, p50, p84, and GAPDH

p65

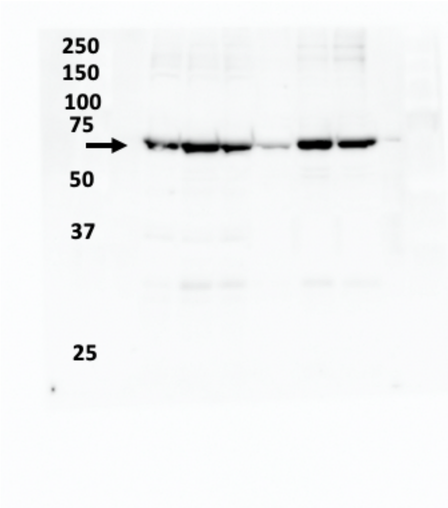

p50

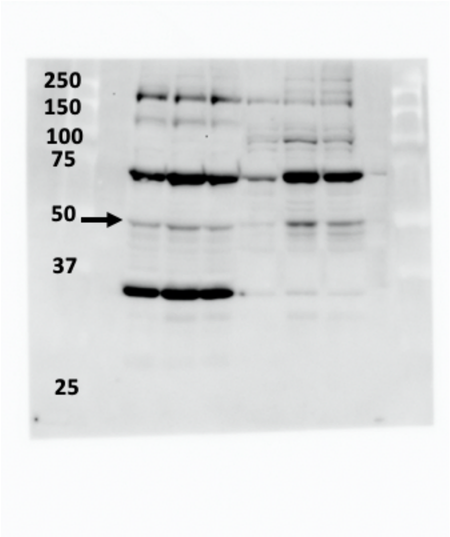

p84

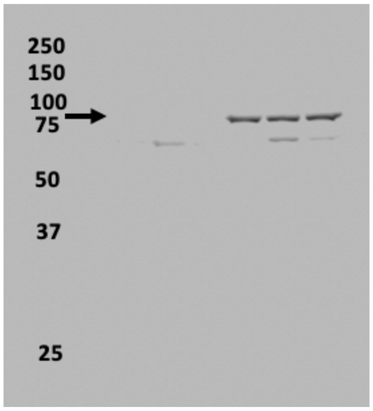

White light

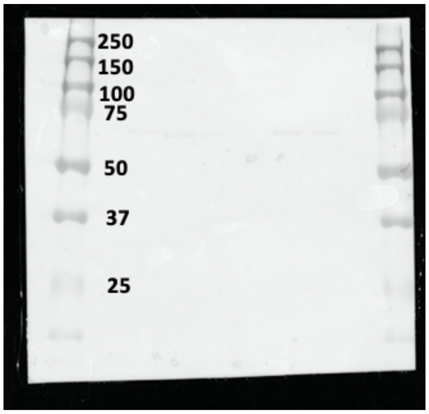

GAPDH

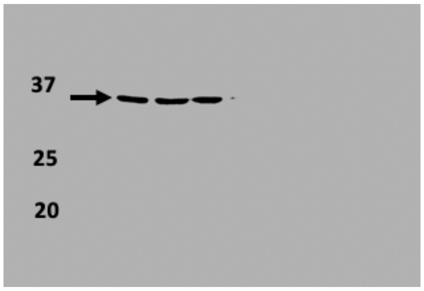

White light

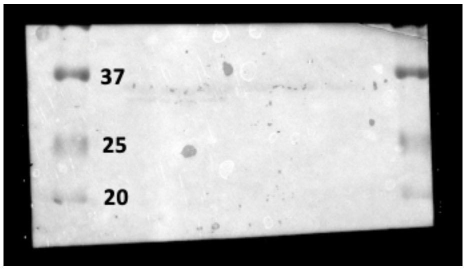

Source data for Supplementary Figure S7: CD47 and GAPDH

CD47

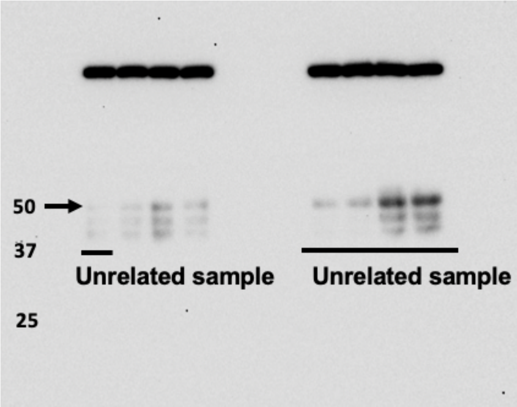

GAPDH

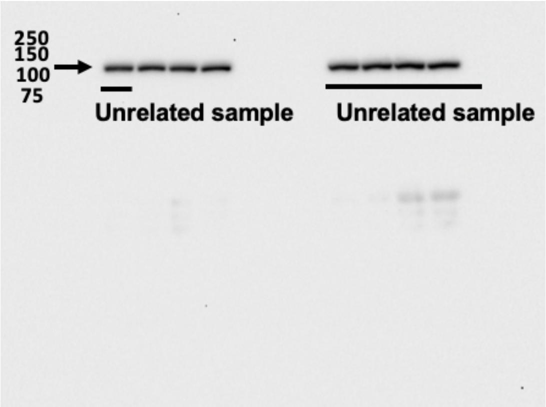

White light

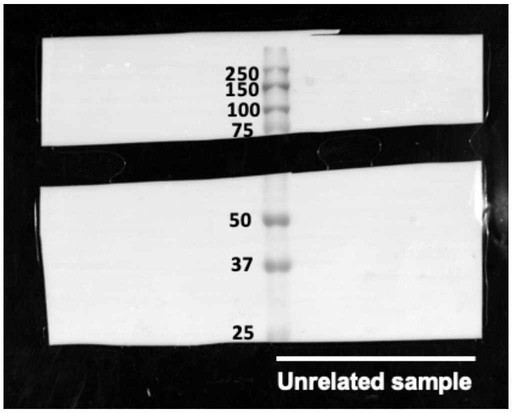

Supplementary Table S1 Online: List of primers used in this study

Primers used with SYBR Green		
**Gene names**	**Direction**	**Primer sequences (5'-3')**
hCD31	Forward	GCAACACAGTCCAGATAGTCGT
	Reverse	GACCTCAAACTGGGCATCAT
hVCAM	Forward	TAACGGGGAGCTACAGCC
	Reverse	CAGCCTGGTTAATTCCTTCAC
hVEGFR2	Forward	ACAGCCTCTGCCAATCCATG
	Reverse	AAGGATGCATTCTTAAGCTCC
hICAM	Forward	GGCCGGCCAGCTTATACAC
	Reverse	TAGACACTTGAGCTCGGGCA
hCyclinD1	Forward	AGCTCCTGTGCTGCGAAGTGG
	Reverse	GGTGTAGATGCACAGCTTCTC
hPCNA	Forward	TGGAGAACTTGGAAATGGAAAC
	Reverse	GAACTGGTTCATTCATCTCTATGG
hC-Myc	Forward	CACCAGCAGGGACTCTGA
	Reverse	GATCCAGACTCTGACCTTTTG
hCyclinA2	Forward	GAGGACCAGGAGAATATCAAC
	Reverse	AGCCAGGGCATCTTCACGCTC
hGAPDH	Forward	AGCCACATCGCTCAGACAC
	Reverse	GCCCAATACGACCAAATCC
mTNF-α	Forward	TCAGGTTGCCTCTGTCTCAG
	Reverse	GCTCTGTGAGGAAGGCTGTG
mIL-1β	Forward	CACAGCAGCACATCAACAAG
	Reverse	GTGCTCATGTCCTCATCCTG
mIL-6	Forward	GCTACCAAACTGGATATAATC
	Reverse	CCAGGTAGCTATGGTACTCCA
mINOS	Forward	CAGCTGGGCTGTACAAACCTT
	Reverse	CATTGGAAGTGAAGCGGTTCG
mENOS	Forward	CCTCGAGTAAAGAACTGGGAAGTG
	Reverse	AACTTCCTTGGAAACACCAGGG
mTNAP	Forward	CGGATCCTGACCAAAAACC
	Reverse	TCATGATGTCCGTGGTCAAT
mNFATC1	Forward	CCTCGAACCCTATCGAGTGT
	Reverse	TCTGTGCTCTGCTTCTCCAC
mCTSK	Forward	CTCCATCGACTATCGAAAGAAAG
	Reverse	AAAGCCCAACAGGAACCAC
mACP5	Forward	CGTCTCTGCACAGATTGCAT
	Reverse	AAGCGCAAACGGTAGTAAGG
mMMP9	Forward	ACGACATAGACGGCATCCA
	Reverse	GCTGTGGTTCAGTTGTGGTG
mGAPDH	Forward	AGCTTGTCATCAACGGGAAG
	Reverse	TTTGATGTTAGTGGGGTCTCG

Primers used with TaqMan probe	
**Gene names**	**Primer ID**
hCD47	Hs00179953_m1
hGAPDH	Hs99999905_m1
mCD47	Mm00495011_m1
mGAPDH	Mm99999915_g1
